# Effect of Membrane Permeance and System Parameters on the Removal of Protein-Bound Uremic Toxins in Hemodialysis

**DOI:** 10.1007/s10439-023-03397-6

**Published:** 2023-11-22

**Authors:** Chun Man Chow, Aaron H. Persad, Rohit Karnik

**Affiliations:** 1 Department of Chemical Engineering, Massachusetts Institute of Technology, 25 Ames St, Cambridge, MA 02142 USA; 2 Department of Mechanical Engineering, Massachusetts Institute of Technology, 77 Massachusetts Ave, Cambridge, MA 02139 USA

**Keywords:** Chronic kidney disease, Nanoporous graphene, Dialysis, Mass transfer, Modeling, Indoxyl sulfate, *p*-cresyl sulfate

## Abstract

Inadequate clearance of protein-bound uremic toxins (PBUTs) during dialysis is associated with morbidities in chronic kidney disease patients. The development of high-permeance membranes made from materials such as graphene raises the question whether they could enable the design of dialyzers with improved PBUT clearance. Here, we develop device-level and multi-compartment (body) system-level models that account for PBUT-albumin binding (specifically indoxyl sulfate and *p*-cresyl sulfate) and diffusive and convective transport of toxins to investigate how the overall membrane permeance (or area) and system parameters including flow rates and ultrafiltration affect PBUT clearance in hemodialysis. Our simulation results indicate that, in contrast to urea clearance, PBUT clearance in current dialyzers is mass-transfer limited: Assuming that the membrane resistance is dominant, raising PBUT permeance from 3 × 10^−6^ to 10^−5^ m s^−1^ (or equivalently, 3.3 × increase in membrane area from ~ 2 to ~ 6 m^2^) increases PBUT removal by 48% (from 22 to 33%, i.e., ~ 0.15 to ~ 0.22 g per session), whereas increasing dialysate flow rates or adding adsorptive species have no substantial impact on PBUT removal unless permeance is above ~ 10^−5^ m s^−1^. Our results guide the future development of membranes, dialyzers, and operational parameters that could enhance PBUT clearance and improve patient outcomes.

**Supplementary Information:**

The online version contains supplementary material available at 10.1007/s10439-023-03397-6.

## Introduction

Chronic kidney disease (CKD) accounts for more than 1 million deaths worldwide annually and is one of the top 20 leading causes of years of life lost [49]. In its extremes, CKD leads to premature mortality due to cardiovascular disease or end-stage kidney disease (ESKD). As the main form of treatment for ESKD patients, hemodialysis plays a crucial role in patient survival by (1) removing excess fluid in the blood stream that has accumulated in the patient, and (2) clearing wastes/toxins while retaining essential proteins. During hemodialysis, blood and dialysate (a saline solution that matches plasma composition to prevent the loss of key minerals) flow counter-current in a dialyzer, separated by a semi-permeable membrane. The toxins move across pores in the membrane by molecular diffusion across a concentration gradient and by convection (i.e., ultrafiltration) induced by a small pressure gradient, whereas larger proteins such as albumin are retained by the membrane mainly through a size-sieving mechanism [35]. Typically, hemodialysis is performed 3 times a week, for 4 h per session [37].

Historically, dialysis focused on removing small, water-soluble toxins such as urea and creatinine, and thus traditional dialyzers and membranes were designed and optimized to effectively remove these compounds [18]. Urea removal, as quantified by the urea reduction ratio and Kt/V (with K being the dialyzer urea clearance rate expressed as volume per unit time, t the dialysis time, and V the volume of water in a patient’s body), is still used as the main reference to describe dialysis performance [3, 10]. However, growing evidence over the last three decades points to the adverse impacts of another class of toxins that were originally overlooked, namely protein-bound uremic toxins (PBUTs). They are relatively more hydrophobic molecules that bind tightly to serum albumin, typically at Sudlow’s sites [46]. PBUTs are generated primarily in the digestive tract of CKD patients due to a modified microbiome and biochemical environment, and accumulate in the body because of the patients’ compromised renal metabolism/transport and inadequate removal by traditional dialysis sessions and dialyzers that are designed to retain albumin [18]. Accumulation of PBUTs in the body leads to higher rates of renal failure and cardiovascular damage through mechanisms including glomerular sclerosis, reactive oxygen species generation, endothelial dysfunction, and defective leukocyte adhesion [23, 47]. To date, more than 30 PBUTs have been reported in the European Uremic Toxin Work Group’s database, out of which indoxyl sulfate (IS) and *p*-cresyl sulfate (pCS) are two of the most well-studied PBUTs that exhibit renal and cardiovascular toxicity [23]. Enhancing PBUT removal in dialysis can thus have a significant impact on reducing patient morbidity and mortality.

The increased recognition and understanding of PBUTs’ toxicity have spurred efforts to address the PBUT problem and move beyond urea-based metrics [3]. Apart from reducing PBUT production through dietary changes or probiotics/prebiotics/synbiotics, four kinds of methods have been explored (computationally or experimentally and with/without patients) to improve PBUT removal during hemodialysis [45, 28, 53]. (1) The earliest proposed methods are simple to implement, involving changing the operational parameters of existing dialysis systems, such as (1a) adjusting the blood or dialysate flow rates [31], or (1b) longer treatment times. Despite their simplicity, the former is not as effective (the reason will be clarified in this paper), and the latter might not be desirable from the patients’ standpoint [2]. (2) The development of high-flux membranes with larger pores enabled hemodiafiltration, which involves increasing ultrafiltration to increase toxin removal [28, 44]. While effective, this method requires a larger volume of dialysis fluid and could potentially increase albumin removal which, in excess, can negatively impact patients’ health [21, 22, 40]. In addition to flow modifications, some emerging ideas that have been shown to improve toxin removal include: (3) Displacement or adsorption-based mechanisms, where (3a) binding competitors are introduced into the blood stream to displace PBUTs, or (3b) adsorbents such as charcoal, albumin, or liposomes are added to the dialysate side or the membrane [5, 26, 38]. However, the former involves introducing foreign substances into the patient; the latter was originally designed primarily for liver disease patients as conventional hemodialysis fails to improve liver detoxification, but is shown to be inadequate for PBUT removal [45, 52]. (4) Membrane improvement, where structural modifications or introduction of bioengineered tubular cells on the membrane raise the selectivity and/or mass transfer rate, without necessarily requiring changes in flow rates [54].

The use of additional fluid or materials in (2) or (3) increases the complexity of the process, could potentially lead to complications in the patients, and may drive up operational cost. Improving flow (1) or the membrane (4), in contrast, does not involve changing the current device “circuit” or introducing other agents into the system. Since hollow-fiber membrane dialyzers that are commonly used today rely more on diffusive than convective transport for toxin removal, especially for PBUTs, increasing membrane permeance has been recognized as a remedy for poor toxin removal. Although significant work, including improving polymer recipes and manufacturing strategies to yield narrower pore size distributions with larger pores and sharper size cutoffs, has been pursued, these enhancements are limited by the structures and thicknesses (≥ 35 μm for wall, ~ 50 nm to 1 μm selective ‘skin’ layer) that have been achieved with the current polymeric membranes [35]. Hence, the majority of the PBUT removal studies have focused on strategies (1-3) that do not involve altering the membrane [45].

The emergence of new membrane materials opens the possibility to surpass these limitations and achieve higher membrane permeance with better control of pore characteristics. For example, nanoporous atomically thin membranes (NATMs) made from graphene have been shown both experimentally and computationally to permit rapid solute diffusion while maintaining selectivity, with the selective layer being only 1-atom (< 0.4 nm) thick, and membrane fabrication using scalable methods has been demonstrated [4, 7, 8, 19, 20, 12]. Single-layer graphene membranes can also withstand pressure differentials up to 100 bar if placed on the appropriate support structure, compared to typical transmembrane pressure (TMP) of < 300 mmHg (0.4 bar) [35, 50]. However, the impact of using high-permeance membranes for dialysis, and whether substantial increase in membrane permeance can enhance PBUT *vs.* non-protein-bound uremic toxin removal, have not been fully investigated. Early computational studies of PBUT removal by hemodialysis used simplified models of mass transfer in the dialyzer to understand the effect of parameters such as dialyzer flow rate or the dialyzer mass transfer coefficient, without modeling processes in the body [30–32]. Clinical studies, sometimes accompanied by similar models, experimentally measured the effect of parameters such as dialysate flow rate, or dialyzer mass transfer coefficient by connecting two dialyzers in series, and quantified the accompanying increase in PBUT clearance [25, 31]. More recently, Maheshwari et al. integrated body compartment models and dialyzer mass transfer models to examine PBUT removal performances of different modes of dialysis (hemodialysis, hemodiafiltration, membrane adsorption, binding competition) [28, 27]. These studies typically examined one or two specific parameters, which makes it difficult to get an overview of the various operational regimes and to identify when each PBUT improvement strategy could be more useful. To further improve toxin removal in hemodialysis, it is important to identify the strategies that offer the greatest benefit, and to examine when different strategies can act synergistically. Furthermore, many studies take the dialyzer geometry and hemodialysis process parameters to be given, but it is unclear whether they are optimal, particularly for high-permeance membranes. A more comprehensive picture of the dialyzer operation is therefore needed, holistically examining the effects of multiple parameters including the membrane permeance.

In this study, to deconvolute the dialyzer performance from the dialyzer-body-system performance where previous literature focused on, we first used a transport-kinetics model to identify the current operating regime in conventional hemodialysis and quantify the extent to which increasing membrane permeance *vs.* other strategies (e.g., flow adjustments, adsorption) can enhance removal of both PBUT and non-PBUT toxins in the dialyzer. We then considered the interaction between the dialyzer and the body through a multi-compartment model to analyze how higher permeance enhances PBUT removal in the overall dialysis process. The study aimed to understand the parameter space at the dialyzer level and the effect of process parameters on PBUT removal during hemodialysis, with the goal of guiding hemodialysis dialyzer, membrane, and process design to improve the patient quality of life.

## Methods

**Fig. 1:**
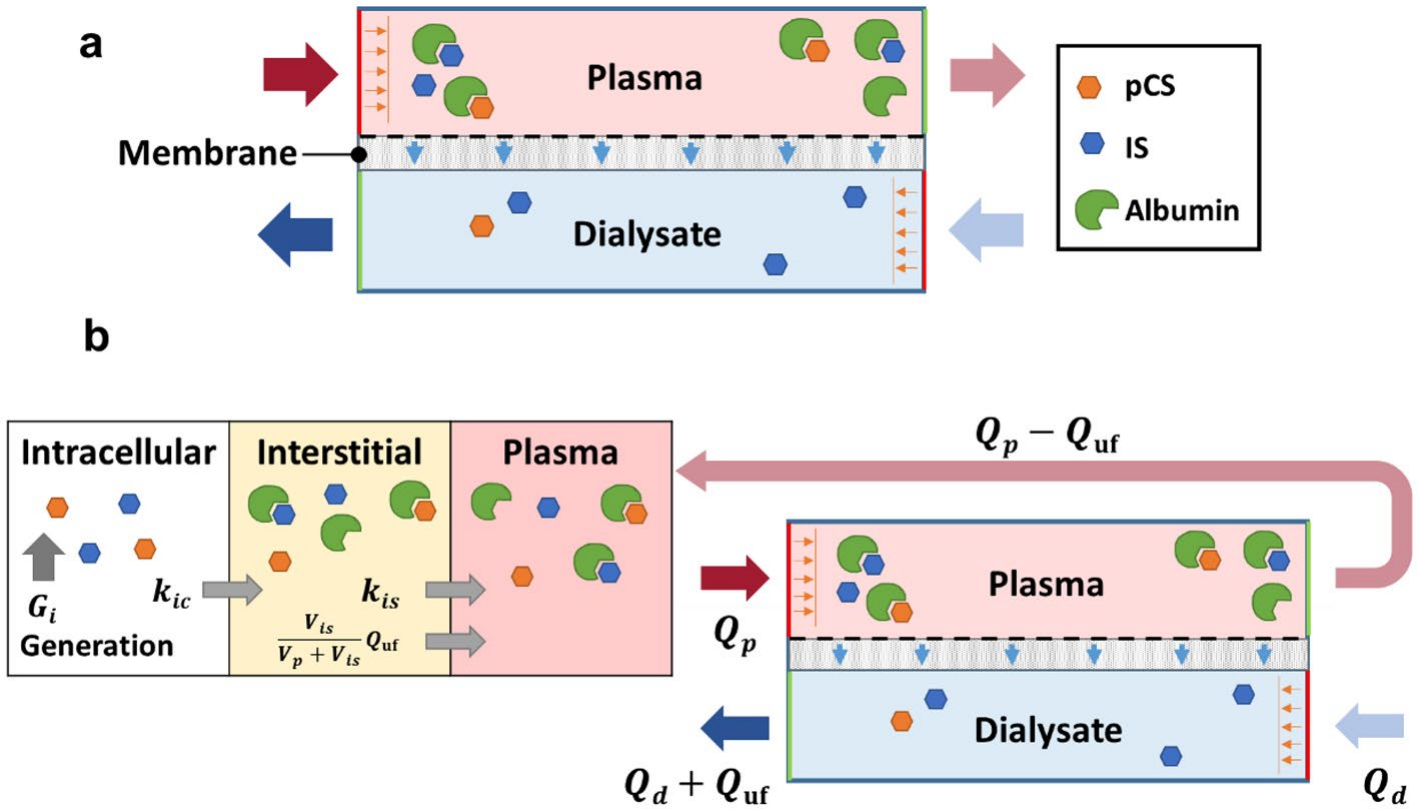
Schematics for the **a** device and **b** compartment models used in this study

To identify gaps in current hemodialysis devices and opportunities to improve PBUT removal, we constructed two models to describe the dynamics of toxin association/dissociation with proteins and the diffusive and convective transport of the toxins across the membrane. The first is a dialyzer device-level model that considers the counter-current flow of plasma and dialysate separated by a membrane (Fig. 1a), and the second system-level model connects the device model to three compartments (intracellular, interstitial, plasma) that serve as proxies to describe toxin generation and partition in the human body (Fig. 1b) [28, 27, 31, 48]. Although our models were derived differently, the resulting equations capture the same transport and kinetics phenomena and are consistent with those of Maheshwari et al. [28, 27], which have been validated against clinical data [13, 15].

### Device Model

Briefly, the one-dimensional (1D) device model accounts for the protein-toxin interactions within the counter-flowing plasma and dialysate channels, considering convection (ultrafiltration) and diffusive transport of toxins across the membrane. For simplicity, a uniform ultrafiltration velocity throughout the channel was used to capture the convection. The case for non-uniform ultrafiltration is described in Supplementary Material S1. Diffusive transport of each toxin $i$ is described by an overall permeance ${P}_{\text{df},i}$, defined as the diffusive mass flux ${J}_{i}$ divided by the concentration difference of the free toxin between the blood and dialysate, $({c}_{p,i}-{c}_{d,i})$, and can be determined using the membrane permeance ${P}_{\text{m},i}$ and the bulk boundary layer resistances ${k}_{p,i}^{-1}$ and ${k}_{d,i}^{-1}$ in the absence of net ultrafiltration [35, 55].

1 $$J_{i} = P_{{{\text{df}},i}} \left( {c_{p,i} - c_{d,i} } \right)$$

2 $$P_{{{\text{df}},i}}^{ - 1} = P_{\text{m},i}^{ - 1} + k_{p,i}^{ - 1} + k_{d,i}^{ - 1}$$

Convective transport of toxins (due to ultrafiltration) is nonlinearly added to this diffusive transport by solving a 1D mass transport equation in the direction perpendicular to the membrane, resulting in a general molecular flux expression that is a function of concentrations in the blood and dialysate at each position along the channel (Supplementary Material S1). The boundary layer resistances were assumed to be small relative to the membrane resistance. The extreme limit of very high permeance of 10^−4^ m s^−1^ considered in this study (for a representative PBUT diffusivity on the order of ~ 10^−9^ m^2^s^−1^) [34] corresponds to a maximum diffusive boundary layer thickness of ~ 10 μm, which, although not well-studied, could potentially be achieved in channel heights on the order of 100 μm since the tumbling of cells, along with engineered mixing features, could promote mixing [29, 42]. For example, Marschewski et al. demonstrated that addition of herringbone features in a microchannel could enhance heat transfer compared to a plain channel, with Nusselt numbers of 18 and 37 at Reynolds numbers of 190 and 510, respectively, corresponding to effective boundary layer thicknesses of 14.8 μm and 7.3 μm [29]. If the boundary layer resistance is significant, the results still hold provided the boundary layer resistance and the membrane resistance are added in series to calculate the overall permeance (Supplementary Material S3). These assumptions allow for a 1D representation of hemodialysis flow in the dialyzer typically assumed in literature [28, 27, 48].

The model considers two common PBUTs, indoxyl sulfate (IS) and *p*-cresyl sulfate (pCS), and describes their competitive binding with the protein albumin (P). Two cases were considered: (1) kinetics: using rate constants to describe the second-order association and first-order dissociation, and (2) equilibrium: assuming equilibrium between the toxins and albumin at each point in the channel, described by equilibrium constants. Previous literature had considered both cases, but there has not been clear indication on when the equilibrium assumption is valid *vs*. the more general kinetic expressions [28, 27, 31, 48]. Comparing equilibrium *vs.* non-equilibrium transport in a direction normal to the membrane allowed us to identify that binding/unbinding kinetics in the boundary layer should be considered only when the boundary layer resistance dominates over the membrane resistance (Supplementary Material S3). We further validated the 1D flow and local equilibrium assumption used in this study by demonstrating that, for the given kinetics parameters, reasonable boundary layer thicknesses, and permeances examined, the concentrations within the boundary layers did not deviate substantially from the bulk values or from equilibrium, and both kinetics and equilibrium models gave identical results for all cases examined (Supplementary Material S3–S4). We therefore performed our simulations with the more general (and also faster) kinetics approach, except when substantial amount of protein was introduced to the dialysate, which was more readily solved using the equilibrium model. The behavior of non-protein-bound toxins was modeled separately without considering binding to albumin. Detailed model equation derivation and discussions on the simulation and key assumptions are included in Supplementary Material S1–S4.

Equations (3–8) are the final dimensionless equations for the kinetics model describing the mass balance of species $i$ (toxins: Z, protein: P, protein-toxin complexes: PZ) in the blood/plasma ($p$) or dialysate ($d$) channels of the device (3–4), the bulk reaction kinetics in compartment $j = p, d$ (5), the overall mass transfer across the membrane (6), and the bulk flow rates (7–8) (see Tables 1 and 2 for variable definitions; tilde denotes dimensionless parameters):

3 $$\frac{{\partial \tilde{c}_{p,i} }}{{\partial \tilde{t}}} = - \frac{\partial }{{\partial \tilde{x}}}\left[ {\tilde{u}_{p} \tilde{c}_{p,i} } \right] - \text{Pe}_{\text{uf}}^{ - 1} g_{i} \left( {\tilde{x}} \right) + \tilde{R}_{p,i}$$

4 $$\frac{{\partial \tilde{c}_{d,i} }}{{\partial \tilde{t}}} = + \frac{\partial }{{\partial \tilde{x}}}\left[ {\tilde{u}_{d} \tilde{c}_{d,i} } \right] + \frac{{h_{p} }}{{h_{d} }}\text{Pe}_{\text{uf}}^{ - 1} g_{i} \left( {\tilde{x}} \right) + \tilde{R}_{d,i}$$

5 $$\tilde{R}_{j,i} = \left\{ {\begin{array}{*{20}c} { - {\text{Da}}_{{\text{Z}}} \tilde{c}_{{j,{\text{Z}}}} \tilde{c}_{{j,{\text{P}}}} + {\text{Da}}_{{ - {\text{Z}}}} \tilde{c}_{{j,{\text{PZ}}}} } & { i = {\text{Z}}} \\ { - \mathop \sum \limits_{{\text{Z}}} {\text{Da}}_{{\text{Z}}} \tilde{c}_{{j,{\text{Z}}}} \tilde{c}_{{j,{\text{P}}}} + {\text{Da}}_{{ - {\text{Z}}}} \tilde{c}_{{j,{\text{PZ}}}} } & {i = {\text{P}}} \\ {{\text{Da}}_{{\text{Z}}} \tilde{c}_{{j,{\text{Z}}}} \tilde{c}_{{j,{\text{P}}}} - {\text{Da}}_{{ - {\text{Z}}}} \tilde{c}_{{j,{\text{PZ}}}} } & { i = {\text{PZ}}} \\ \end{array} } \right.\quad j = p,d$$

6 $$g_{i} \left( {\tilde{x}} \right) = \left\{ {\begin{array}{*{20}c} {\frac{{S_{\infty ,i} \left( {\tilde{c}_{p,i} - \tilde{c}_{d,i} } \right)}}{{{\text{Pe}}_{{{\text{m}},i}} }}} & & {{\text{Pe}}_{{{\text{m}},i}} \ll 1} \\ {S_{\infty ,i} \tilde{c}_{p,i} } & & {{\text{Pe}}_{{{\text{m}},i}} \gg 1} \\ {\frac{{S_{\infty ,i} \left( {e^{{{\text{Pe}}_{{{\text{m}},i}} }} \tilde{c}_{p,i} - \tilde{c}_{d,i} } \right)}}{{e^{{{\text{Pe}}_{{{\text{m}},i}} }} - 1}}} & & {{\text{otherwise}}} \\ \end{array} } \right.,\;{\text{where}}\;{\text{Pe}}_{{{\text{m}},i}} = S_{\infty ,i} {\text{Pe}}_{{{\text{uf}}}}^{ - 1} /{\text{Pe}}_{{{\text{df}},i}}^{ - 1}$$

7 $$\tilde{u}_{p} \left( {\tilde{x}} \right) = 1 - \text{Pe}_{\text{uf}}^{ - 1} \tilde{x}$$

8 $$\tilde{u}_{d} \left( {\tilde{x}} \right) = \frac{{h_{p} }}{{h_{d} }}\left[ {\tilde{Q}_{d/p} + \text{Pe}_{\text{uf}}^{ - 1} \left( {1 - \tilde{x}} \right)} \right]$$

**Table 1:** Dimensionless parameters governing the hemodialysis device system

Type	Parameter	Expression^a^	Description	Base value
Geometric ratio	$({h}_{p}/{h}_{d})$	$\frac{{h}_{p}}{{h}_{d}}$	Plasma *vs*. dialysate channel geometric ratio ($h$ = channel volume per membrane area, e.g., channel height for a rectangular cross-section channel)	0.853
Flow rate ratio	${\widetilde{Q}}_{d/p}$	$\frac{{Q}_{d,\text{in}}}{{Q}_{p,\text{in}}}$	Dialysate *vs*. plasma inlet flow rates	4.10
Mass transfer (Inverse Péclet)^b^	${\text{Pe}}_{\text{uf}}^{-1}$	$\frac{{Q}_{\text{uf}}}{{Q}_{p,\text{in}}}$	“Dimensionless ultrafiltration rate”: Membrane ultrafiltration *vs.* channel convection flow rates	5.13 × 10^−2^
	${\text{Pe}}_{\text{df},i}^{-1}$	$\frac{{P}_{\text{df},i}{A}_{\text{m}}}{{Q}_{p,\text{in}}}=\frac{{\left({K}_{o}A\right)}_{i} }{{Q}_{p,\text{in}}}$	“Dimensionless mass transfer coefficient”: Membrane diffusion (permeance) *vs*. channel convection flow	Z = pCS, IS: 0.574 P, PZ: 0
	${S}_{\infty ,i}$	${S}_{\infty ,i}$	Sieving coefficient; 1 - ${\sigma }_{i}$, where ${\sigma }_{i}$ is the reflection coefficient	Z = pCS, IS: 1 P, PZ: 0
Kinetics (Damköhler)^c^	${\text{Da}}_{\text{Z}}$	$\frac{{k}_{\text{Z}} L{c}_{0}}{{u}_{p,\text{in}}}$	Forward (association) reaction rate *vs.* channel convection	pCS: 1.76 × 10^4^ IS: 1.76 × 10^4^
	${\text{Da}}_{-\text{Z}}$	$\frac{{k}_{-\text{Z}} L}{{u}_{p,\text{in}} }=\frac{{\text{Da}}_{\text{Z}}}{{\widetilde{K}}_{\text{Z}}}$ ${\widetilde{K}}_{\text{Z}}={K}_{\text{Z}}{c}_{0}$	Backward (dissociation) reaction rate *vs.* channel convection, where ${K}_{\text{Z}}$ is the equilibrium constant [M^−1^], i.e., the ratio between forward/backward reaction rates	pCS: 5.02 × 10^2^ IS: 5.13 × 10^2^

^a^ Volumetric flow rates are used since they are typically the relevant operational parameters. One can also define the parameters using flow velocities, i.e., ${\widetilde{Q}}_{d/p}=({u}_{d,\text{in}}{h}_{d})/({u}_{p,\text{in}}{h}_{p})$, ${\text{Pe}}_{\text{uf}}^{-1}=({v}_{\text{uf}}/{u}_{p,\text{in}})(L/{h}_{p})$, ${\text{Pe}}_{\text{df},i}^{-1}=({P}_{\text{df},i}/{u}_{p,\text{in}}) (L/{h}_{p})$.

^b^ Conventionally, Péclet number is a ratio comparing convective/diffusive flux. Here, we define the “inverse Péclet” such that the channel convection rate is in the denominator and ultrafiltration or diffusion rate is in the numerator to ease comparison. Membrane literature often uses a membrane Péclet number ${\text{Pe}}_{\text{m},i}={S}_{\infty ,i}{\text{Pe}}_{\text{uf}}^{-1}/{\text{Pe}}_{\text{df},i}^{-1}={S}_{\infty ,i}{v}_{\text{uf}}/{P}_{\text{df},i}={S}_{\infty ,i}{Q}_{\text{uf}}/{\left({K}_{o}A\right)}_{i}$ to compare 1D convective (ultrafiltration) and diffusive fluxes of the solute across the membrane, where KoA = ${P}_{\text{df}}{A}_{\text{m}}$ is the dialyzer mass transfer area coefficient.

^c^ ${c}_{0}$ is the concentration scale, set as the total (bound and unbound) inlet protein concentration (Supplementary Material S3b).

**Table 2:** Key variables and parameters in the model (see Supplementary Material S3 for determination of parameter values)

Type	Symbol	Quantity	Units	Base value	Source
Simulation	${c}_{j,i}$	Concentration on blood (plasma)/ dialysate ($j$* =* $p$,$d$) side of species $i$	M	–	–
	$t$	Time, non-dimensionalised by $L/{u}_{p,\text{in}}$	s	–	–
	$x$	Position along dialyzer length, non-dimensionalized by $L$	cm	–	–
Dialyzer geometry	${A}_{\text{m}}$	Membrane area	m^2^	1.87	[28]
	${h}_{p}$	Blood (plasma)/ dialysate channel volume per membrane area, i.e., channel height for a rectangular system; calculated from fixed membrane area and channel cross-sectional area	μm	52.5	–
	${h}_{d}$			61.5	–
	$L$	Dialyzer length	cm	23	[28]
	$w$	Membrane area to dialyzer length ratio, equivalent to 2 $\pi RN$, where fiber radius $R$ = 105 μm, and number of fibers $N$ = 12300	m	8.11	[28]
Dialyzer bulk flow	${Q}_{p,\text{in}}$	Plasma/dialysate inlet bulk channel volumetric flow rate (blood inlet flow of 300 mL min^−1^ with 35% hematocrit)	mL min^−1^	195	[28]
	${Q}_{d,\text{in}}$			800	[28]
	${u}_{p,\text{in}}$	Plasma/dialysate inlet bulk channel flow rate (speed)	cm s^−1^	0.763	[28]
	${u}_{d,\text{in}}$			2.67	[28]
Membrane flux	${Q}_{\text{uf}}$	Total ultrafiltration flow rate	mL min^−1^	10	[28]
	${v}_{\text{uf}}$	Ultrafiltration velocity	m s^−1^	8.93 × 10^−8^	–
	${S}_{\infty ,i}$	Sieving coefficient; 1 - ${\sigma }_{i}$, where ${\sigma }_{i}$ is the reflection coefficient	–	≈1 (pCS,IS) 0 (protein)	–
	${P}_{\text{df},i}$	Overall permeance for toxins	m s^−1^	3 × 10^−6^	S3
Reaction	${k}_{\text{A},\text{B}}$	Forward reaction rate for A, B	M^−1^ s^−1^	1.67 × 10^6^	[21]
	${K}_{\text{A}}$	Equilibrium constant for A (pCS)	M^−1^	1.00 × 10^5^	[12]
	${K}_{\text{B}}$	Equilibrium constant for B (IS)	M^−1^	0.98 × 10^5^	[12]
Compartment model	${G}_{i}$	Generation rate	mg min^−1^	0.02557 (pCS), 0.02477 (IS)	[28]
	${k}_{{\varvec{i}}{\varvec{c}},i}$, ${k}_{is,i}$	Free toxin mass transfer coefficient (intracellular to interstitial, interstitial to plasma), same for pCS, IS	mL min^−1^	100, 1135	[28]
	${V}_{ic}$, ${V}_{is}$,${V}_{p}$	Compartment volume (intracellular, interstitial, plasma)	L	28, 12, 3.5	[28]

Table 1 lists the key dimensionless parameters that govern the system behavior. The parameters are often expressed as ratios that compare the different geometrical terms or flux/reaction rates in the dialyzer, and represent the relative strengths/time scales of each process. For instance, ${\text{Pe}}_{\text{df},Z}^{-1}\gg {\text{Pe}}_{\text{uf}}^{-1}$ based on baseline values listed in the table suggests that diffusion is the dominant transport mechanism compared to ultrafiltration in a typical hemodialysis dialyzer.

Simulation parameter values were drawn from literature, with base values obtained from typical dialyzer geometry and dialysis operation settings (Table 2; Supplementary Material S3) [28, 27, 51]. The dialyzer geometry is defined by the membrane area, channel cross-sectional area, and dialyzer length. Species concentrations were solved in space using an ordinary differential equation (ODE) solver in MATLAB and the shooting method (Supplementary Material S2). We examined the effects of changing dialysate flow rate, ultrafiltration rate, overall permeance, and the addition of an albumin adsorbent on the dialysate side on PBUT removal (Table 3).

**Table 3:** Overview of in-silico experiments performed using the two models

	Device model	Compartment model
Model description	Physics-based, steady-state kinetics-mass transfer model describing toxin transport and protein-binding kinetics within a dialyzer with counter-current flows and ultrafiltration	Dynamic pharmacokinetic body-compartment model describing species partitioning and transport in the body over a hemodialysis treatment period, combined with a time-dependent device model
Model assumptions	• 2 PBUTs (IS, pCS) and albumin, or urea alone • PBUT-albumin binding kinetics described by first order dissociation and second order association rate laws • Concentrations described by 1D coordinate along the channels -Kinetics effects are negligible within the membrane/boundary layer (local equilibrium assumption) -Transport across the membrane is driven by both diffusion (overall permeance computable from resistance-in-series model) and convection (uniform ultrafiltration rate) • Transport along the channels is driven by convection alone (i.e., by plasma or dialysate flow; insignificant effect of diffusion)	• 3-compartment model (plasma, interstitial, intracellular) for 2 PBUTs (IS, pCS) and albumin - Fluid removable by ultrafiltration only present in the plasma and interstitial compartments - Constant toxin generation rates in intracellular compartment - Only toxins are transported across the compartments, driven by their concentration gradient • 2-compartment model (plasma, intracellular) for urea • Device model assumptions remain the same
Goal	Understand the effects on toxin removal in the dialyzer by: • Changing overall permeance, dialysate flow rate, and ultrafiltration rate, relative to plasma flow rate (PBUTs and urea) – Fig. 2 • Adding albumin adsorbent in the dialysate (PBUTs only; equilibrium assumed between PBUTs and albumin) – Fig. 3	Examine the impact on PBUT and urea removal in a hemodialysis session by: • Changing overall permeance and dialysate flow rate – Figs. 4, 5 • Changing overall permeance, blood flow rate, membrane area, and dialysis duration – Fig. 6
Key metric for PBUT removal	Device removal ratio (DRR) = steady state single-pass total PBUT removal by dialyzer – Eq. 9	Fractional net removal (${f}_{\Delta {q}_{\text{net}}}$) = Net total PBUT removal from the body by the dialyzer over the dialysis session, normalized by the initial total PBUT mass in the body – Eq. 11

For details, please refer to Supplementary Material S3

We defined the “device removal ratio” for toxin $\text{Z}$ (DRR_Z_) as a performance metric for toxin removal in the device. DRR describes the ratio between the total amount of toxins (bound plus unbound: ${c}_{j,{\text{Z}}_{\text{tot}}}={c}_{j,\text{Z}}+{c}_{j,\text{PZ}}$) removed by the device *vs*. that entering the device, i.e., the single-pass toxin removal:

9 $${\text{DRR}}_{{\text{Z}}} = \frac{{h_{d} }}{{h_{p} }}\frac{{\tilde{u}_{d} \left( {\tilde{x} = 0} \right)}}{{\tilde{u}_{{p,{\text{in}}}} }}\frac{{c_{{d,{\text{Z}}_{{{\text{tot}}}} }} \left( {\tilde{x} = 0} \right)}}{{c_{{p,{\text{Z}}_{{{\text{tot}}}} }} \left( {\tilde{x} = 0} \right)}} = 1 - \frac{{\tilde{u}_{p} \left( {\tilde{x} = 1} \right)}}{{\tilde{u}_{{p,{\text{in}}}} }}\frac{{c_{{p,{\text{Z}}_{{{\text{tot}}}} }} \left( {\tilde{x} = 1} \right)}}{{c_{{p,{\text{Z}}_{{{\text{tot}}}} }} \left( {\tilde{x} = 0} \right)}}$$

To our knowledge, most reported metrics in hemodialysis literature (e.g., reduction ratio or clearance) focus on overall hemodialysis toxin removal, which combines the body and device characteristics, making it hard to compare different devices. In contrast, a metric like “device removal ratio” (DRR) allows the device performance to be characterized independently, which would aid dialyzer design. The DRR characterizes the performance of the dialyzer at a given point in time, as opposed to over the entire dialysis session. DRR = 1 implies that 100% of the total amount of toxin present in the blood stream flowing into the dialyzer is removed before the blood returns to the patient. The rate of toxin removal is given by the blood flow rate multiplied by the blood toxin concentration and DRR.

### Compartment Model

The second model connects the device model to the three compartments (plasma: $pl$, interstitial: $is$, intracellular: $ic$) in the human body to examine PBUT removal over a dialysis session. We investigated the effects of changing overall permeance, blood flow rate, membrane area, and dialysis duration on PBUT removal (Table 3). The model formulation was adapted from previous literature and is described in Supplementary Material S5 [28, 27, 39].

Two metrics were used to quantify the amount of toxin removed from the body during the dialysis session, namely (1) net removal ($\Delta {q}_{\text{net}}$ [g]), which is the total amount of toxin removed over the dialysis duration $\tau$, and can be calculated either from the decrease in total toxin mass within all three compartments plus the amount generated, or by the toxin removed in the dialysate, and (2) fractional net removal (${f}_{\Delta {q}_{\text{net}}}$), which is defined as the net removal normalized by the initial total toxin mass in the body:

10 $$\Delta q_{{{\text{net}}}} = {\Delta }\left[ {c_{{{pl}}} V_{{{pl}}} + c_{{{is}}} V_{{{is}}} + c_{{{ic}}} V_{{{ic}}} } \right]_{{\tau - \left( {t = 0} \right)}} + G\tau = \mathop \smallint \limits_{t = 0}^{\tau } (Q_{d} + Q_{{{\text{uf}}}} )c_{{d,{\text{out}}}} dt$$

11 $$f_{{\Delta q_{{{\text{net}}}} }} = \frac{{\Delta q_{{{\text{net}}}} { }}}{{\left[ {c_{{{pl}}} V_{{{pl}}} + c_{{{is}}} V_{{{is}}} + c_{{{ic}}} V_{{{ic}}} } \right]_{t = 0} }}$$

Note that $\Delta {q}_{\text{net}}$ is identical to the total solute removal (TSR) metric, calculated in some experimental studies by multiplying the dialysate solute concentration in the spent dialysate by the sum of the dialysate volume and ultrafiltration volume [9].

Simulation parameters were drawn from literature (Table 2; Supplementary Material S3). The partial differential equations across time and space were solved using the method of lines, using finite differencing across the membrane length and MATLAB’s ODE solver for time.

## Results

### Dialyzer Performance for PBUT Removal

**Fig. 2:**
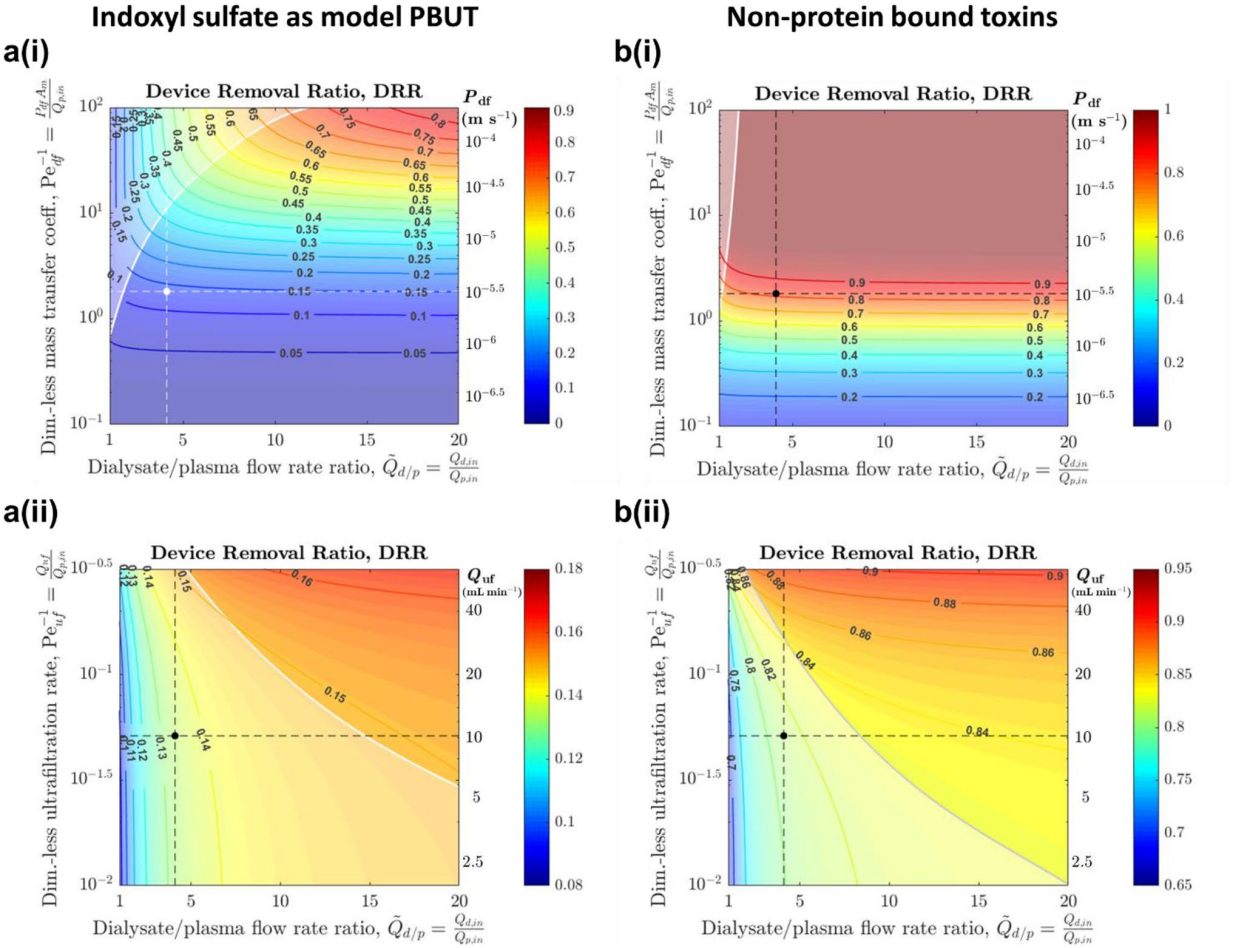
Effect of increasing dialysate flow rate ${Q}_{d,\text{in}}$, overall permeance ${P}_{\text{df}}$, or ultrafiltration rate ${Q}_{\text{uf}}$ on toxin removal for **a** PBUTs and **b** Non-PBUTs, e.g., urea, creatinine. The contour levels and color (note the different scales across sub-figures) denote the device removal ratio (DRR). Dimensionless parameters are plotted. All plots: $x$ = dialysate/plasma flow ratio. Top panels (i): $y$ = ${\text{Pe}}_{\text{df},\text{IS}}^{-1}\propto {P}_{\text{df},\text{IS}}$, constant ${Q}_{\text{uf}}$ = 10 mL min^−1^. Bottom panels (ii): $y$ = ${\text{Pe}}_{\text{uf}}^{-1}\propto$ ${Q}_{\text{uf}}$, constant ${P}_{\text{df},\text{IS}}$ = 3 × 10^−6^ m s^−1^. Baseline parameter levels for IS are denoted by the white/black dot and dashed lines for **a**, and the same dot and lines are presented in **b**. The transition between the mass-transfer-limited (clear) and dialysate-removal-limited (whiter) regimes is also delineated

For the base case scenario reflecting typical hemodialysis, which corresponds to blood and dialysate flow rates of 300 and 800 mL min^−1^, ultrafiltration rate of 10 mL min^−1^, and overall PBUT permeance of 3 × 10^−6^ m s^−1^ (Table 1; Supplementary Material S3), the steady state single-pass toxin removal (DRR) for PBUTs is only 13%, compared to 81% of non-PBUTs for the same permeance (Fig. 2), consistent with literature observations that PBUT clearance in dialysis sessions are limited [26, 43, 53]. The high DRR for non-PBUTs indicates that the dialyzer is not the limiting factor for clearance of toxins that are similar in size or smaller than the model PBUTs (including urea), since nearly all of the toxin flowing into the dialyzer is removed. However, the low DRR for PBUTs indicates that the dialyzer does not adequately remove the PBUTs, and supports the observation that strong protein binding severely impedes toxin clearance if PBUT transport across the body compartments is not a limiting factor [25, 30, 51]. Due to the similar kinetic parameters for IS and pCS which give almost identical concentration profiles (Supplementary Material S6), we selected IS as the model PBUT and results are discussed in terms of IS removal, although the model framework is applicable for other PBUTs.

To quantitatively assess the extent to which different strategies can enhance toxin removal for a typical hemodialysis device, the key dimensionless parameters for dialysate flow rate ${\widetilde{Q}}_{d/p}$, ultrafiltration rate ${\text{Pe}}_{\text{uf}}^{-1}$, and overall permeance ${\text{Pe}}_{\text{df},i}^{-1}$ (see Table 1) were varied and the corresponding DRR was determined (Fig. 2; dashed lines indicate the base operating condition). We expect that increasing the dialysate flow rate (${\widetilde{Q}}_{d/p}$), ultrafiltration rate (${\text{Pe}}_{\text{uf}}^{-1}$), and overall permeance (${\text{Pe}}_{\text{df},i}^{-1}$) would improve DRR through diluting the dialysate stream to maximize the concentration gradient, increasing ultrafiltration flux, and increasing diffusive flux, respectively.

We did not examine the effect of varying Damköhler numbers since in all cases considered in this study, the PBUT-albumin binding/unbinding kinetics are extremely fast and thus inconsequential. The Damköhler number for dissociation of PBUTs from albumin is 500 in the base case, and the plasma residence time in the dialyzer will need to be smaller by two orders of magnitude before the kinetics become significant in affecting PBUT removal.

Increasing the dialysate to blood flow ratio ${\widetilde{Q}}_{d/p}$, i.e., moving horizontally rightward on Fig. 2a(i), is equivalent to flushing the dialysate side to remove toxins more quickly. At the estimated base levels of ${\widetilde{Q}}_{d/p}$ and permeance, only a small amount of PBUTs enter the dialysate due to the high mass transfer resistance and the limited concentration of the free (unbound) toxin; thus increasing ${\widetilde{Q}}_{d/p}$ alone has minimal impact on DRR. In contrast, increasing the dimensionless mass transfer coefficient ${\text{Pe}}_{\text{df},i}^{-1}$ (moving vertically upward and increasing the KoA, either by (i) increasing the membrane permeance, or by (ii) increasing membrane area), significantly improves the DRR up to an order of magnitude increase in ${\text{Pe}}_{\text{df},i}^{-1}$, beyond which the DRR saturates since toxin removal is limited by the dialysate flow rate. These trends in dialysate flow rates and KoA are consistent with observations in experimental, modeling, and clinical studies [25, 27, 31, 32].

Similarly, Fig. 2a(ii) examines the effect of changing the dialysate to plasma flow rate ${\widetilde{Q}}_{d/p}$ and the ultrafiltration rate ${\text{Pe}}_{\text{uf}}^{-1}$. Increasing both ${\widetilde{Q}}_{d/p}$ and ${\text{Pe}}_{\text{uf}}^{-1}$ would improve PBUT removal, although the increase in DRR is significantly smaller in our model since the ultrafiltration rates examined were constrained based on what is reasonable in practice. This is because conventional hemodialysis poses a limit on the amount of fluid that can be removed from the patient, unless additional fluid is supplied but would require sterile replacement fluid to be infused and could reduce diffusive flux by diluting the blood stream (Supplementary Material S3c). Similar low gain in PBUT removal by increasing ultrafiltration rates was also shown in previous literature [22, 32].

The results show that, for typical ultrafiltration rates in hemodialysis that have limited effect on PBUT clearance (as discussed above), the hemodialysis process can be separated into two regimes based on DRR: a mass-transfer-limited regime where changing dialysate flow parameters does not significantly affect the DRR, and a dialysate-removal-limited regime where changing permeance has little effect on the DRR (denoted by the whiter regions). Figure 2a(i) indicates that current devices are deep within the mass-transfer-limited regime. Overall, the results in Fig. 2a provide strong evidence that illustrate the potential of high-permeance membranes (or larger membrane areas) in improving PBUT removal.

Non-protein bound toxins, such as urea and creatinine, also exhibit the above regimes, but the regimes are shifted with respect to the dimensionless permeance and dialysate flow rate since clearance is not hindered by binding to albumin (Fig. 2b). For non-PBUTs that are similar in size or smaller than IS, existing dialyzers effectively remove most of the toxin (DRR is close to 1), and increasing neither the permeance nor the dialysate flow rate has a large effect on the DRR, a finding that is also observed in prior studies [25, 31]. It is noteworthy that, while increasing blood flow rate has been shown to improve toxin removal during hemodialysis treatment (due to the higher amount of blood processed) [27], it tends to reduce the DRR through decreasing ${\widetilde{Q}}_{d/p}$, ${\text{Pe}}_{\text{df},i}^{-1}$, and ${\text{Pe}}_{\text{uf}}^{-1}$, and therefore toxin removal in the dialyzer does not increase proportionally with the blood flow rate.

**Fig. 3:**
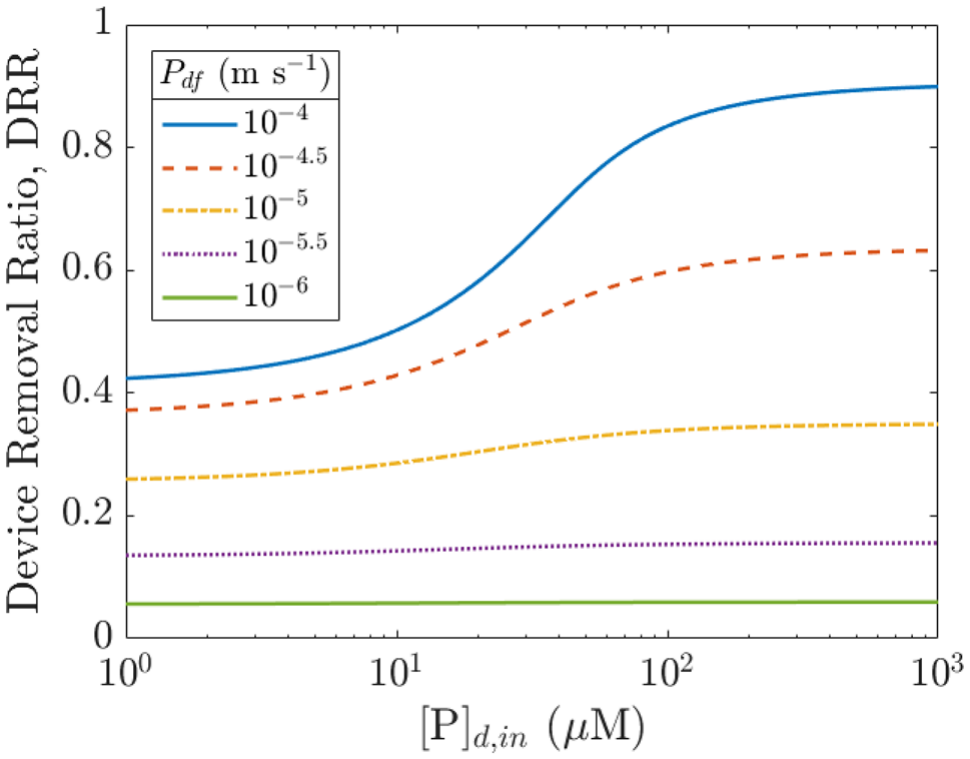
Effect of adding adsorbent (albumin protein, P) to the dialysate inlet (expressed as albumin concentration) on PBUT removal at various permeances as predicted by the equilibrium model. Typical albumin concentration in blood is ~ 600 μM

We also explored the addition of an adsorbent (albumin, 10^0^–10^3^ μM; typical blood concentration ~ 600 μM) [6] to the dialysate as a strategy for PBUT removal. Adsorbents (e.g., albumin, activated carbon, β-cyclodextrin) [5, 41] could increase diffusive flux by reducing the free toxin concentration in the dialysate due to their high adsorption affinity. Introducing albumin adsorbents on the dialysate side has no notable effect on PBUT removal at low permeance, since the adsorbent affects removal via a mechanism analogous to increasing dialysate flow rate. The adsorbent only starts to play a role when permeance is sufficiently high (> 1 × 10^−5^ m s^−1^) where sufficient amount of toxin begins to be transported across the membrane to affect the concentration gradient (Fig. 3).

### PBUT Removal from Patients During a Dialysis Session

To examine how the insights gained from the device model can be translated to practical hemodialysis operation, we turned to the multi-compartment model, which describes toxin (IS, pCS) generation and partitioning in the human body in addition to the mass transfer and kinetics in the device. In the model, toxins are produced in the intracellular compartment, which is connected to the interstitial compartment that is linked to the plasma compartment. Blood is drawn from the plasma compartment into the device’s plasma channel, and flows back into the body (Fig. 1b). The governing parameters in addition to those listed in Table 1 that describe the process include the toxin generation rates, the mass transfer rates between compartments, and their distribution volumes, given in Table 2 [27, 28]. The device geometry and flows were kept the same as in the device model. For better interpretability of results, we use dimensional parameters in this section.

**Fig. 4:**
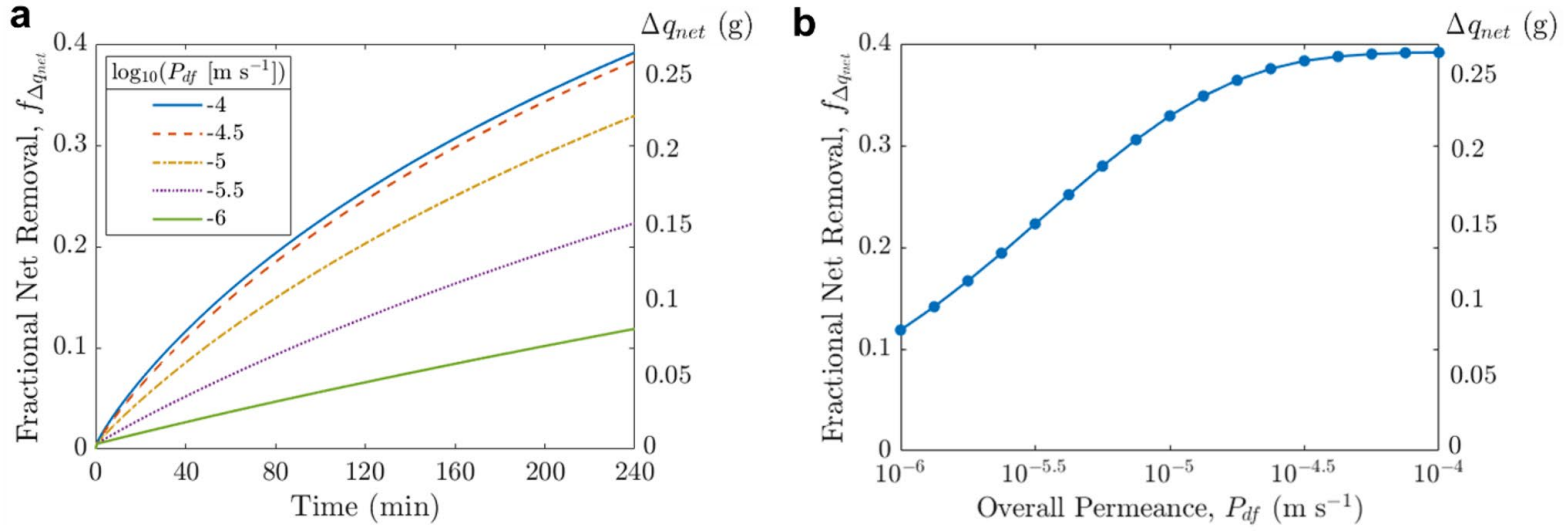
**a** PBUT fractional net removal (indoxyl sulfate) over time during a typical dialysis session as estimated using the multi-compartment model, with parameters set to be same as the device model (${Q}_{b,\text{in}}$, ${Q}_{d,\text{in}}$, ${Q}_{\text{uf}}$ = 300, 800, 10 mL min^−1^). The body initially contains ~ 0.66 g of IS. Net removal, ∆*q*_net_, is also presented on the right axis. **b** Removal performance at 4 h of dialysis as a function of overall permeance. Performance with other metrics is included in Supplementary Material S7

The compartment model was used to estimate dialyzer performance over a typical dialysis session of $\tau$ = 4 h with membrane area ${A}_{\text{m}}$ = 1.87 m^2^ and blood flow rate ${Q}_{b}$ = 300 mL min^−1^ (base case scenario), for overall permeance to IS ranging from 10^−6^ to 10^−4^ m s^−1^ (Fig. 4). Common metrics used to describe toxin removal include clearance (${K}_{cl}$, mL min^−1^), reduction ratio (RR), and net removal ($\Delta {q}_{\text{net}}$ [g]; Eq. (10)) [28]. In this study, we focused on net removal and fractional net removal (${f}_{\Delta {q}_{\text{net}}}$, Eq. (11)) because net removal is a direct metric that measures how much toxin is removed inside the human body over time, whereas ${K}_{cl}$ and RR are proxy indicators for the toxin removed that were originally introduced to describe systems with no protein binding and fewer compartments. Supplementary Material S7 defines and discusses the metrics in more detail.

Typical IS permeance ${P}_{\text{df}}$ is around 3 × 10^−6^ = 10^−5.5^ m s^−1^, which corresponds to ${f}_{\Delta {q}_{\text{net}}}$ of 22% (${K}_{cl}$ ~ 19 mL min^−1^, RR ~ 29%, $\Delta {q}_{\text{net}}$ ~ 0.15 g) for the base system in our compartment model. The PBUT clearance numbers are consistent with reported values in multiple literature studies [14, 28, 53]. Raising permeance by half an order of magnitude from 3 × 10^−6^ to 1 × 10^−5^ m s^−1^ can raise net removal substantially from 22 to 33% for the base case scenario. Further increase in permeance faces diminishing returns in PBUT removal and saturates beyond 3 × 10^−5^ m s^−1^, after which removal is limited by the toxin concentration in the dialysate stream. The effect of permeance on overall removal as indicated by the fractional net removal (${f}_{\Delta {q}_{\text{net}}}$) appears smaller than the often-reported reduction ratio (RR) since it takes time for PBUTs to move from the intracellular to the blood compartment, and RR is biased toward removal from the plasma as it depends only on the ratio of final and initial plasma toxin concentrations (Supplementary Material S7).

**Fig. 5:**
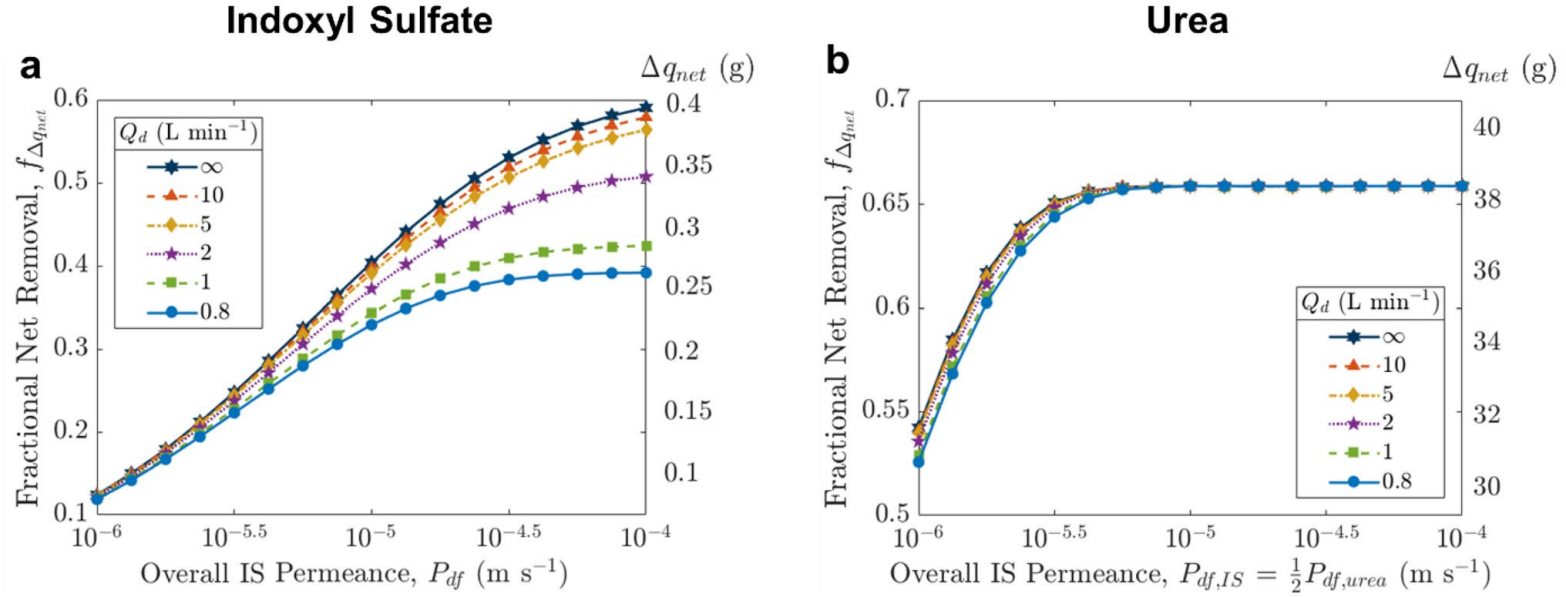
**a** Effect of dialysate flow rate on fractional net removal (indoxyl sulfate) during a typical dialysis session for various permeance values. ∞ refers to the limiting case of infinitely high dialysate flow rate. **b** Same plot for urea, assuming ${P}_{\text{df},\text{urea}}$ = 2 ${P}_{\text{df},\text{IS}}$ based on the ratio of diffusivities of urea and IS

Similar to the device model, we examined the effect of dialysate-based strategies on toxin removal for various permeance levels, where the maximum possible removal was obtained by setting infinite dialysate flow rate (or zero toxin concentration in the dialysate, corresponding to high adsorbent concentration) (Fig. 5a). As observed in the case of steady state device operation, the dialysate flow rate (or addition of adsorbent) is relevant only at higher permeance (${P}_{\text{df}}$ > 10^−5^ m s^−1^) as the system moves away from the mass-transfer-limited regime. The highest fractional net removal of ~ 0.6 occurs at high permeance and high dialysate flow rates, where removal is limited by the transport of PBUT out of the intracellular compartment. The results again demonstrate the significant enhancement achievable via improving diffusive mass transfer by increasing the membrane permeance or area that allows synergistic dialysate-based strategies to become effective.

For comparison with IS removal, we also studied urea removal using a two-compartment model (Supplementary Material 8). We assumed urea permeance to be twice that of indoxyl sulfate based on the ratio of their diffusivities, and verified the baseline results against literature, i.e., urea reduction ratio ~ 0.65 and clearance ~ 186 mL min^−1^ (Fig. 5b; Supplementary Material S8). Due to the lack of protein binding and higher intra/extracellular mass transfer coefficient, removal is significantly higher for urea *vs.* PBUTs. Urea removal is almost the same across all ${Q}_{d}$ rates examined, and DRR approaches 1 at permeance ${P}_{\text{df},\text{urea}}$ > 1 × 10^−5^ m s^−1^ (Fig. 2b(i)), meaning that nearly all the urea that enters the dialyzer is removed. The system is then limited by the amount of urea able to enter the device during treatment, as observed by many and suggested by the commonly used ballpark metric, Kt/V = ${Q}_{b}\tau /V$, where V is the urea distribution volume [35].

### Parameter Space for Hemodialysis with High-Permeance (or Large-Area) Membranes

**Fig. 6:**
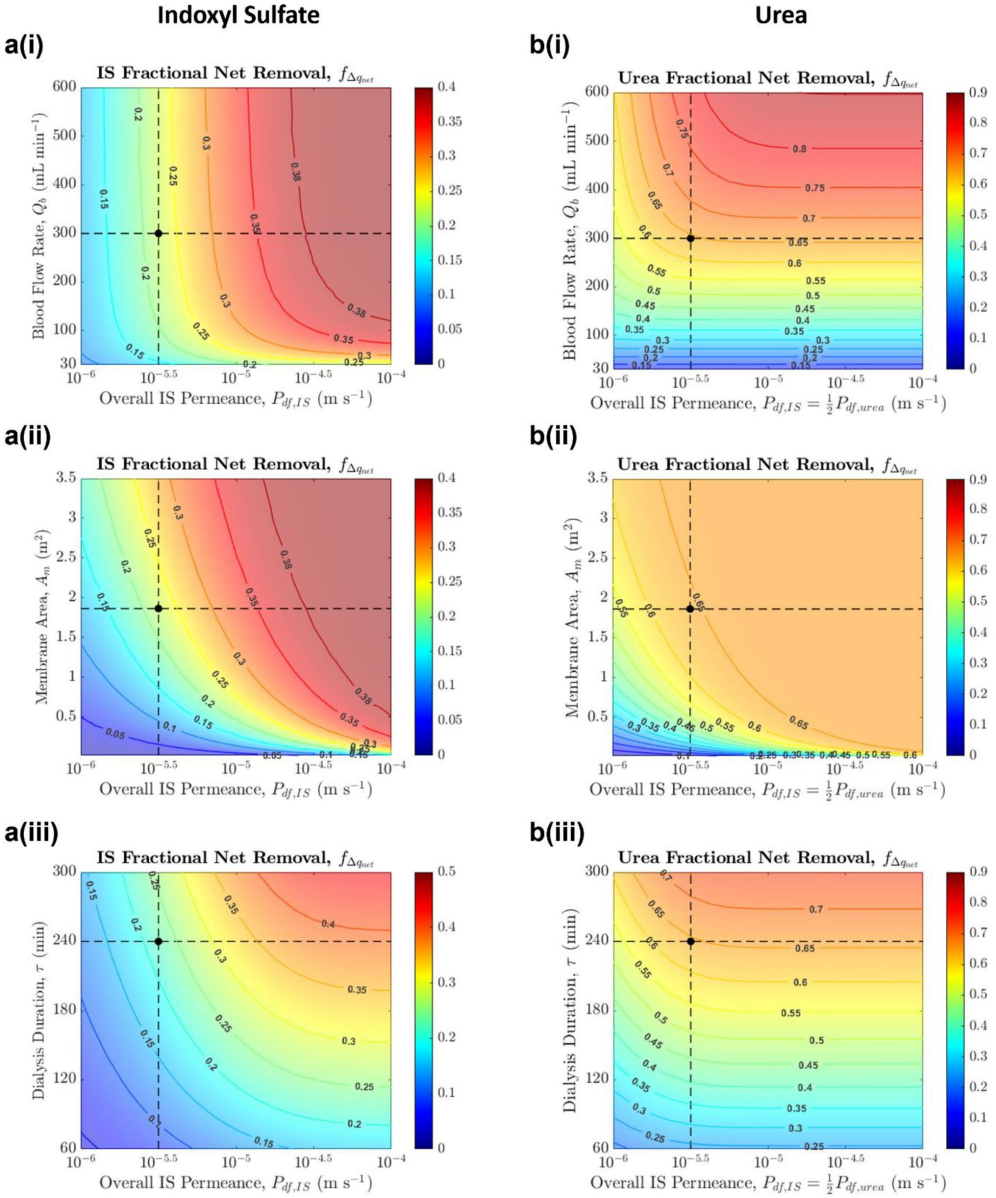
Effect of changing the overall permeance of **a** indoxyl sulfate and **b** urea and (i) blood flow rate, (ii) membrane area, (iii) dialysis duration simultaneously on fractional net removal, while keeping other parameters constant (${V}_{\text{uf}}$ = 2.4 L). Base case conditions: ${A}_{\text{m}}$ = 1.87 m^2^, ${Q}_{b}$ = 300 mL min^−1^, $\tau$ = 4 h, ${P}_{\text{df},\text{IS}}$ = 3 × 10^−6^ m s^−1^ (black dot, dashed lines). Note the different scales for fractional net removal across sub-figures

We now turn to the implications of higher membrane permeance on the hemodialysis process (required blood flow rate ${Q}_{b}$, dialysis duration $\tau$) or the dialyzer size (membrane area ${A}_{\text{m}}$). Figure 6 shows how variations in permeance and ${Q}_{b}$, ${A}_{\text{m}}$, or $\tau$, while holding other parameters constant at the typical operating conditions (${Q}_{b}$ = 300 mL min^−1^, ${A}_{\text{m}}$ = 1.87 m^2^, $\tau$ = 4 h, ultrafiltration volume *V*_uf_ = 2.4 L), affect toxin removal.

At typical blood flow rates of 300 mL min^−1^, increasing ${Q}_{b}$ (moving upwards in Fig. 6a(i)) does not enhance PBUT removal significantly, as observed in experimental studies [27]. The reason is that slow toxin transport out of the intracellular compartment and limited toxin transfer across the membrane lead to a relatively uniform PBUT concentration on the blood side along the length of the dialyzer; increasing ${Q}_{b}$ therefore has little effect on the PBUT concentration difference across the membrane and hence little effect on PBUT removal. Unlike ${Q}_{b}$, changing membrane area and dialysis duration affects PBUT removal substantially (Fig. 6a(ii–iii)). In fact, for a given net removal, permeance and ${A}_{\text{m}}$ hold an inverse relationship as the two appear together in the governing dialyzer equations as ${P}_{\text{df}}{A}_{\text{m}}= {K}_{o}A$, the typically reported dialyzer mass transfer-area coefficient. Increasing membrane area (through increasing the fiber perimeter or number of layers/bundles) will yield the same effect as improving permeance, though the need to maintain a small membrane module size makes the latter somewhat more practical. The observation that increasing KoA increases PBUT clearance is consistent with previous clinical research [25].

Figure 6a shows that increasing permeance leads to higher IS removal, or, it can enable some reduction in membrane area, flow rate, or dialysis time without compromising PBUT removal. In contrast to IS, Fig. 6b illustrates that increasing permeance (equally for PBUT and urea) or ${A}_{\text{m}}$ would not lead to much improvement in urea removal, and decreasing ${Q}_{b}$ and $\tau$ hurts urea clearance quite substantially, as urea removal is limited by how much urea can enter the dialyzer.

The preceding analysis shows that toxin removal is affected by the interactions between multiple parameters. Therefore, providing a means for practitioners to visualize the trade-off between the key operating parameters would be informative in optimizing them. This can be accomplished using a potential map that could offer a sense of the possible operational parameter space to achieve a target toxin removal.

**Fig. 7:**
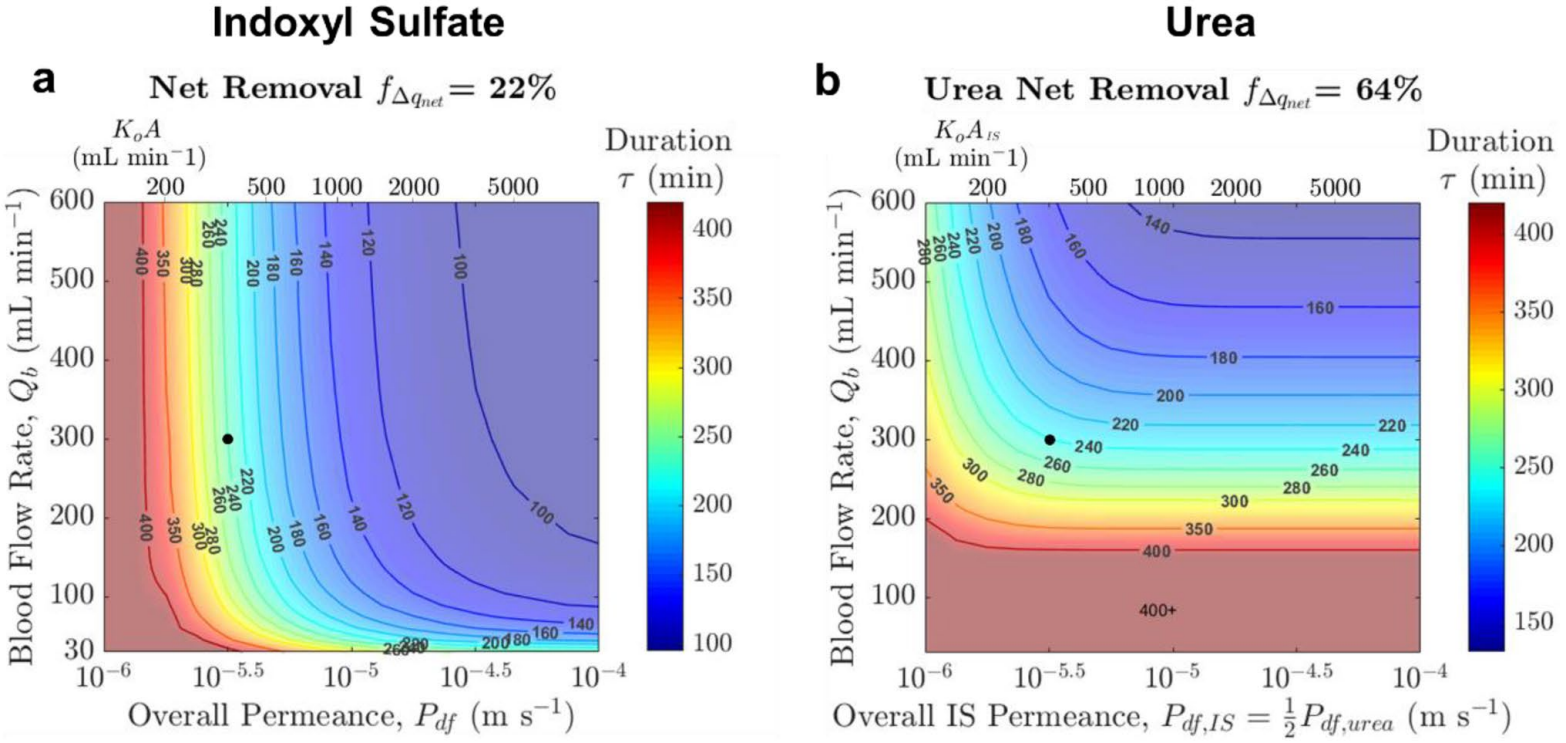
Operational parameter space to meet a hypothetical targeted **a** indoxyl sulfate net removal of 22%, **b** urea net removal of 64%. $x$: Permeance ${P}_{\text{df}}$, or overall mass transfer coefficient ${K}_{o}A$, assuming 1.87 m^2^ membrane area; $y$: Blood flow rate; $z$ (contours): Dialysis duration. The black dots denote the base case conditions

Figure 7 shows all combinations of {${Q}_{b}$, ${P}_{\text{df},\text{IS}}{A}_{\text{m}}$ = ${K}_{o}A$, $\tau \}$ that result in a hypothetical targeted 22% fractional net removal for IS and 64% for urea, with all other parameters held constant at values listed in Table 2. Similar potential maps could be generated for relevant toxin removal targets for different patient needs. For instance, given the current dialyzer geometry and considering IS removal alone, if permeance is raised 10-fold from 3 × 10^−6^ to 3 × 10^−5^ m s^−1^, one can simultaneously decrease ${Q}_{b}$ from 300 to 100 mL min^−1^, $\tau$ from 4 to 3 h, ${A}_{\text{m}}$ from 1.87 to 0.59 m^2^ (factor of 3.16 = 10^0.5^) and still be able to maintain 22% IS net removal (Fig. 7a). However, practical operation would necessitate maintaining adequate urea removal as well. Since urea removal prefers reduction in area over blood flow rate and dialysis duration, the combination of ${Q}_{b}$ = 100 mL min^−1^, ${P}_{\text{df},\text{IS}}$ = 3 × 10^−5^ m s^−1^, ${A}_{\text{m}}$ = 0.59 m^2^ would actually require $\tau$ > 400 min to keep urea removal at 64% (Fig. 7b). Hence, unless the patient can withstand higher levels of urea or there exist strategies to enhance urea removal or control its accumulation [17], an increase in permeance would enable some reduction in ${A}_{\text{m}}$ and only relatively small reduction in ${Q}_{b}$ and $\tau$. Ultimately, the desirable parameter set will be selected depending on the relative clinical importance of various toxins, patient comfort, technical difficulty, material/cost constraint, etc., and more clinical studies are needed to better understand PBUT toxicology and the benefits/disadvantages of adjusting blood flow, treatment time, and membrane area.

## Discussion

Results from the device model demonstrate that the most effective way to enhance PBUT removal in hemodialysis is through increasing the overall mass transfer in the dialyzer, i.e., increasing KoA. For a given membrane area, this can be achieved through increasing the diffusive permeance. Dialysate interventions, e.g., increasing dialysate flow rate or introducing adsorbents, start to have an effect only when permeance to PBUTs reaches 10^−5^ m s^−1^. These results are consistent with the findings of prior experimental and clinical studies, which showed that increasing KoA and/or increasing dialysate flow rates lead to improved PBUT clearance [25, 28, 31, 32]. Our work builds upon these prior works to develop a much more generalizable map of the dialyzer operation with delineation of different mass transfer regimes, and demonstrates quantitatively the difference between PBUT and urea removal. The results illuminate the opportunity in using highly permeable (or larger area) membranes to help toxin removal. For instance, a permeance of ~ 10^−5^ m s^−1^ for a molecule (*L*-tryptophan) similar in size and structure to indoxyl sulfate has already been demonstrated using nanoporous graphene membranes [19], and additional optimization could potentially yield further enhancements. In addition to high permeance for enhanced PBUT clearance, these new membranes should also control albumin loss through careful engineering and ensure adequate mass transfer in the blood and dialysate channels (Supplementary Material S9).

Using the multi-compartment model, we quantified the extent to which such membranes could reduce the toxin levels in the body. We examined how increasing permeance and maintaining the same PBUT removal could allow for reduction in membrane area, required blood flow rate, or dialysis duration, and offered an example visualization potential map that enables clinicians to pick the relevant set of operating parameters for a given target toxin removal to meet the needs of different patients.

On first glance, shorter dialysis duration (not too short to cause a shock/disequilibrium) [36] and lower blood flow rate required might be desirable. Particularly, lower blood flow rate would lower the stress on patient and would also inadvertently increase the dialysate/blood flow rate ratio which improve device PBUT removal in a synergistic way. However, cross-examination against urea removal suggests that these two benefits cannot be realized without significantly compromising urea clearance. In contrast, having smaller membranes could lead to smaller devices, which can be desirable both economically and sustainably as these modules are typically only used once, and are also more suitable for wearable, portable, or implantable dialysis systems [11, 16]. Thus, the emergence of highly permeable membranes in forms such as nanoporous atomically thin membranes not only has the potential to improve the toxin removal capability of hemodialysis devices, but also opens up the possibility of altering the design and operation for hemodialysis.

Despite the importance of high permeance illustrated throughout this study, the strong protein-binding nature of PBUTs means that an upper limit on toxin removal exists, unless the equilibrium could be perturbed. Various strategies have been proposed to achieve it, e.g., introducing binding competitors/displacers, which could be promising [26, 28]. The results show that, coupled with high-permeance membranes, these strategies can synergistically improve PBUT clearance, making it possible that PBUTs can be removed just as effectively in the future as the removal of small uremic toxins achieved today. However, the effect of increasing membrane permeance to high ${P}_{m}$ values of ~ 10^−4^ m s^−1^ will likely be limited due to boundary layer resistances, especially on the plasma side. Thus, the impact of increasing membrane permeance beyond ~ 10^−5^ m s^−1^ will be contingent on the effectiveness of strategies such as engineering features to reduce boundary layer resistances and thus enable enhanced PBUT removal [29]. Increasing the membrane area may be more practical in such cases since it does not face the limitations of boundary layer resistance.

Our study is based on modeling results, and faces several limitations, which include various assumptions and simplifications on bulk mass transfer resistance, protein-binding kinetics, operation conditions, and toxin transport and interactions in the body due to limited available data in the literature and the possibility that the parameters could also vary from patient to patient. Because this study focuses on the dialyzer, we adopted a relatively simple yet data-verified body compartment model [28, 27]. Recent advances in pharmacokinetic modeling indicate that blood regional transport effect for metabolites that interact with proteins could be more complex than that captured by our model. However, our method allows for extension of the body compartment model to include these effects in the future, and could be easily modified as our understanding improves and more information about PBUTs becomes available. For instance, other PBUTs of interest, e.g., 3-carboxy-4-methyl-5-propyl-2-furanpropanoic acid (CMPF), could be added to examine how different dialysis parameters affect their removal, as long as their binding kinetics are known [33, 51]. Nonetheless, our models produce results that are consistent with studies reported in the literature, e.g., capturing quantitatively the relationship between increases in dialyzer mass transfer area coefficient and dialysate flows and the enhancement in toxin removal for both urea and PBUTs [1, 22, 24, 25, 31, 32]. Further clinical studies could validate the insights generated through our models.

In conclusion, this study illustrates that current dialyzers operate in a mass-transfer-limited regime for PBUT removal, in contrast to the blood flow rate-limited regime for urea removal. The findings highlight the importance of improving overall mass transfer through raising membrane permeance or area in increasing PBUT removal, provided that dialyzers are engineered such that mass transfer resistances in the flow channels are not limiting. With the development of novel membrane materials and advances in their manufacturing, such highly permeable membranes are no longer “materials of the future” and their incorporation into hemodialysis devices would have tremendous impact on the lives of kidney patients. Furthermore, the study demonstrates the importance of decoupling device performance from the device-body system to better understand toxin removal in the dialyzer. An enhanced understanding of the operational parameter space around PBUT removal opens up the opportunity to rethink dialysis treatment and the membrane module design, moving beyond the standard form of hemodialysis developed in the 1970s and leading to improved clinical outcomes.

### Supplementary Information

Below is the link to the electronic supplementary material.

Supplementary file1 (DOCX 3148 kb)
