# Supplementary material for: Effect of Membrane Permeance and System Parameters on the Removal of Protein-Bound Uremic Toxins in Hemodialysis
Source: Ann Biomed Eng. 2023 Nov 22;52(3):526–41. doi: 10.1007/s10439-023-03397-6 (PMC10859350; doi:10.1007/s10439-023-03397-6)
Supplement: Supplementary file 1 — Supplementary file1 (DOCX 3148 kb) [file 10439_2023_3397_MOESM1_ESM.docx]

**Effect of membrane permeance and system parameters on the removal of protein-bound uremic toxins in hemodialysis**

**Supplementary Material**

Chun Man Chow,^1^ Aaron H. Persad,^2^ Rohit Karnik^2^

^1^ Department of Chemical Engineering, Massachusetts Institute of Technology, 25 Ames St, Cambridge, MA 02142, USA; ^2^ Department of Mechanical Engineering, Massachusetts Institute of Technology, 77 Massachusetts Ave, Cambridge, MA 02139, USA

[S1. Device model specification: Governing equation derivation 2](#_Toc138234138)

[S2. Simulation 9](#_Toc138234139)

[S3. Model assumptions and observations 10](#_Toc138234140)

[S4. Kinetics versus equilibrium 14](#_Toc138234141)

[S5. Compartment model specification 15](#_Toc138234142)

[S6. Comparison between pCS and IS results 18](#_Toc138234143)

[S7. Removal metrics 19](#_Toc138234144)

[S8. Urea compartment model 24](#_Toc138234145)

[S9. Maximum allowable albumin loss: Implications on protein permeance and selectivity 26](#_Toc138234146)

[S10. Additional device model figures 28](#_Toc138234147)

[S11. References 30](#_Toc138234148)

# S1. Device model specification: Governing equation derivation

The dialysis module is modeled as a one-dimensional system with two inlets (for dialysate and blood/plasma) and two outlets, with the two streams in counter-flow. We use subscript $j=p$ or $d$ to denote plasma (blood) side or dialysate side, and $i$ to denote the species.^6,31^ Without loss of generality, we use $N_{i}$ to express the molecular flux across the membrane [mol s^-1^ m^-2^] and $R_{j,i}$ to express the volumetric reaction rate [mol m^-3^ s^-1^]. Mole balance for an infinitesimal cross-sectional volume $dV_{j}$ in the channel, given a differential membrane area $dA_{m}=w dx$, yields:

$\frac{\partial\left( {dV}_{j}c_{j,i} \right)}{\partial t}=\pm\left( Q_{j,x}c_{j,i,x}-Q_{j,x+dx}c_{j,i,x+dx} \right)\mp N_{i} w dx+R_{j,i}{dV}_{j}$ (1)

where the top and bottom equations represent $j=p$ or $j=d$ respectively, and $c$ is the bulk mean concentration of the free (unbound) toxin. $Q_{j,x}$ is the absolute volumetric flow rate in channel $j$ at position $x$, i.e. all flow rates/velocities are non-negative and go in the +$x$ direction for blood/plasma side and –$x$ direction for the dialysate. $w$ is the “area per length” of the membrane (total membrane area divided by membrane length), which could be the width of the channel in rectangular geometry or the circumference/perimeter of a fiber geometry ($w=2\pi r$, Figure S1b). Such 1D formulation akin to “flat-plate geometry” is adopted for generalizability and would yield the same result as the typical fiber-based membranes if only the flow rate (no velocity profile) is considered. The extension to a rectangular 2D geometry is relatively straightforward and such results would still be applicable to other geometries after transformation and a correction on the surface curvature.

Assuming a constant cross-sectional area $A_{j}$ allows us to write $dV_{j}=A_{j}dx$ and re-write equation (1), via dividing by $dx$ and $A_{j}$ and taking the limit as $dx$ becomes infinitesimally small, as:

$\frac{\partial c_{j,i}}{\partial t}=\mp\frac{1}{A_{j}}\frac{\partial\left( Q_{j}c_{j,i} \right)}{\partial x}\mp\frac{w}{A_{j}}N_{i}+R_{j,i}$ (2)

where the first term $N_{i}$ (flux) involves transport of toxins across the membrane and the second term $R_{j,i}$ (reaction) involves the association and dissociation of the toxins with the protein (albumin), as described in detail in the following sections.


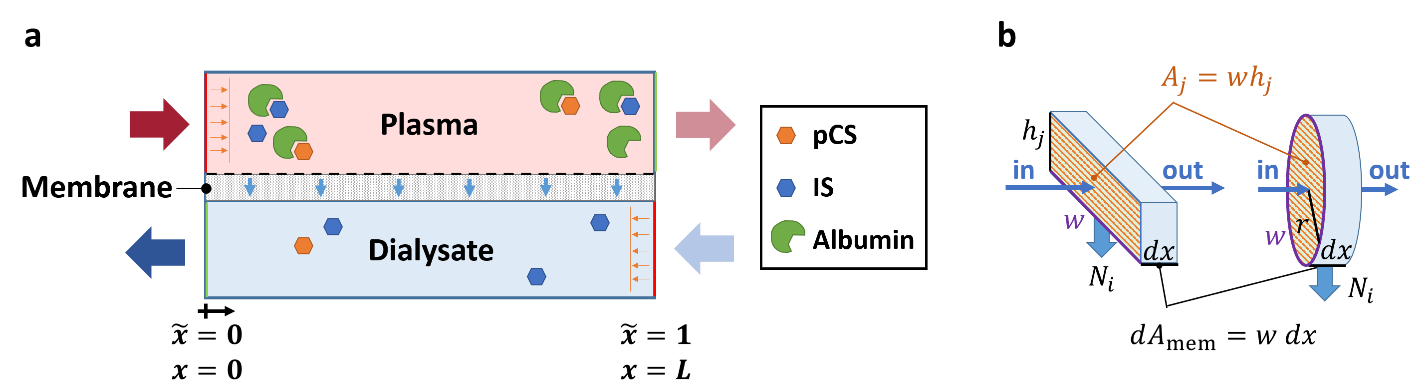


**Figure S****1.** (a) Device model for the dialysis cartridge. (b) Mass balance across an infinitestimally-thin ($dx$) generalized control volume.

*a. Ultrafiltration*

We first consider ultrafiltration, which affects both the bulk flow in the channel and the convective part of the flux term. There are two typical profiles assumed for ultrafiltration along the length of the membrane.^36^ The first assumes a constant ultrafiltration rate, $v_{uf,0}$ [m s^-1^], i.e., equivalent to assuming a uniform transmembrane pressure ($\Delta P$) (Case i); the second assumes a linearly decreasing ultrafiltration rate along the channel, i.e. a linearly decreasing pressure drop (Case ii) (Figure S2). Specifying the ultrafiltration rate profile instead of the pressure simplifies the problem, or else a Navier-Stokes type equation has to be applied to describe the momentum balance. In general, performing steady state mass balance on fluid flow on both sides gives:

$Q_{p}\left( x \right)=Q_{p,\mathrm{in}}-w\int_{0}^{x} v_{\mathrm{uf}}(\hat{x})d\hat{x}$ (3)

$Q_{d}\left( x \right)=Q_{d,\mathrm{in}}+w\int_{x}^{L} v_{\mathrm{uf}}(\hat{x})d\hat{x}$ (4)

where $L$ is the length of the channel and the total volumetric ultrafiltration rate is
$Q_{\mathrm{uf}}=w\int_{0}^{L} v_{\mathrm{uf}}(\hat{x})d\hat{x}$. Note again that the dialysate flow goes in the –$x$ direction.

For (i) constant/uniform ultrafiltration rate, we can write:

$v_{\mathrm{uf}}\left( x \right)=v_{uf,0}$ (5i)

$Q_{p}\left( x \right)=Q_{p,\mathrm{in}}-wv_{uf,0}x$ (6i)

$Q_{d}\left( x \right)=Q_{d,\mathrm{in}}+wL v_{uf,0}\left( 1-\frac{x}{L} \right)$ (7i)

While (i) is not perfectly realistic, it offers a simplified description that still captures the ultrafiltration effect. For (ii) linear ultrafiltration rate we can write:

$v_{\mathrm{uf}}\left( x \right)=v_{uf,min}+\left( v_{uf,max}-v_{uf,min} \right)\left( 1-\frac{x}{L} \right)$ (5iia)

where $v_{uf,min}$ and $v_{uf,max}$ are the lowest and highest ultrafiltration rates at the exit and entrance of the channel respectively.

To make the two assumptions comparable, we define $v_{uf,0}=(v_{uf,max} -v_{uf,min})/2$ as the average change in ultrafiltration rate across the channel, and $\alpha_{\min}=v_{uf,min}/v_{uf,0}$ as the ratio between the minimum and the average change rates. This allows us to re-write equation (5iia) as:

$v_{\mathrm{uf}}\left( x \right)=v_{uf,min}+2v_{uf,0}\left( 1-\frac{x}{L} \right)=v_{uf,0}\left[ \alpha_{\min} +2\left( 1-\frac{x}{L} \right) \right]$ (5iib)

$Q_{p}\left( x \right)=Q_{p,\mathrm{in}}-wv_{uf,0}\int_{0}^{x} \left[ \alpha_{\min} +2\left( 1-\frac{\hat{x}}{L} \right) \right]d\hat{x}=Q_{p,\mathrm{in}}-wv_{uf,0} x\left( \alpha_{\min}+2-\frac{x}{L} \right)$ (6ii)

$Q_{d}\left( x \right)=Q_{d,\mathrm{in}}+wv_{uf,0}\int_{x}^{L} \left[ \alpha_{\min} +2\left( 1-\frac{\hat{x}}{L} \right) \right]d\hat{x}=Q_{d,\mathrm{in}}+wv_{uf,0}L\left( 1-\frac{x}{L} \right)\left( \alpha_{\min}+1-\frac{x}{L} \right)$ (7ii)

To convert from volumetric flow rates in the channel into the corresponding velocities for our 1D system, we set $Q_{j}\left( x \right)=A_{j}u_{j}\left( x \right)=h_{j}wu_{j}\left( x \right)$, where $h_{j}$ is the channel volume per membrane area (height in a rectangular/flat-plate geometry) and $w$ is the membrane “area per length” (width or circumference) of the channels (Figure S1b). We have:

$u_{p}=\frac{Q_{p}(x)}{h_{p}w}=\left\{ \begin{matrix} u_{p,\mathrm{in}}-v_{uf,0}\frac{x}{h_{p}} & (i) \\ u_{p,\mathrm{in}}-v_{uf,0}\frac{x}{h_{p}}\left( \alpha_{\min}+2-\frac{x}{L} \right) & (ii) \end{matrix} \right.$ (8)

$u_{d}=\frac{Q_{d}(x)}{h_{d}w}=\left\{ \begin{matrix} u_{d,\mathrm{in}}+v_{uf,0}\frac{L}{h_{d}}\left( 1-\frac{x}{L} \right) & (i) \\ u_{d,\mathrm{in}}+v_{uf,0}\frac{L}{h_{d}}\left( 1-\frac{x}{L} \right)\left( \alpha_{\min}+1-\frac{x}{L} \right) & (ii) \end{matrix} \right.$ (9)

For simplicity, we proceed with case i in our simulations, though the derivations for both cases are presented below.


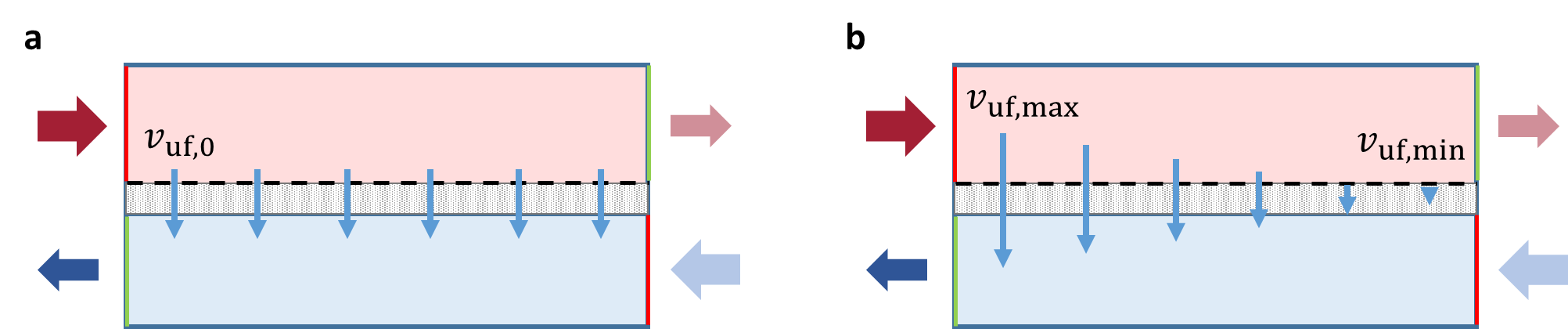


**Figure S****2**. Ultrafiltration rate profiles: (a) constant - Case i, (b) linear - Case ii.^36^

*Non-dimensionalization*

The governing equation (2) was non-dimensionalized by scaling $x$ by the length of the channel, $\tilde{x}=x/L$, the channel velocities by the plasma inlet velocity (after substituting in $Q_{j}=h_{j}wu_{j}$), $\tilde{u}_{j}=u_{j}/u_{p,in}$, time by the plasma channel convective time scale, $\tilde{t}=tu_{p,in}/L$, and concentration by a concentration scale *c*_0_, which was set to be the initial total accessible albumin concentration at the plasma channel inlet (350.9 μM; see section S3b). The variable typeset for concentration is not changed in the equations for clarity:

$\frac{\partial c_{p,i}}{\partial\tilde{t}}=-\frac{\partial\left( \tilde{u}_{p}c_{p,i} \right)}{\partial\tilde{x}}-\frac{L}{h_{p}}\tilde{N}_{i}+\tilde{R}_{p,i}$ (10)

$\frac{\partial c_{d,i}}{\partial\tilde{t}}=+\frac{\partial\left( \tilde{u}_{d}c_{d,i} \right)}{\partial\tilde{x}}+\frac{L}{h_{d}}\tilde{N}_{i}+\tilde{R}_{d,i}$ (11)

where $\tilde{N}_{i}=N_{i}/(u_{p,\mathrm{in}}c_{0})$ is the non-dimensionalized flux and $\tilde{R}_{j,i}=\left( L/u_{p,\mathrm{in}} \right)\left( R_{j,i}/c_{0} \right)$ is the non-dimensionalized volumetric reaction rate. The dimensionless forms of the equations allow us to derive key parameters governing the system (often appear as competitions between different driving forces or as geometric ratios), and the length scales of the system are embedded within these parameters. The first dimensionless group that emerges is a Péclet number comparing the membrane ultrafiltration and channel convection. We define the inverse (based on the convention of membrane flux/bulk convection): $Pe_{uf,0}^{-1}=(v_{\mathrm{uf},0}/h_{p})(L/u_{p,\mathrm{in}})$. We also define a velocity ratio of the dialysate inlet velocity to the plasma inlet velocity: $\alpha_{d/p}=u_{d,\mathrm{in}}/u_{p,\mathrm{in}}$ (equivalent to the flow rate ratio $\tilde{Q}_{d/p}$ = $Q_{d,\mathrm{in}}/Q_{p,\mathrm{in}}$ if the channel heights and widths or perimeters are the same). This allows us to rewrite equations (8-9) for the channel velocities in dimensionless forms:

$\tilde{u}_{p}\left( \tilde{x} \right)=\left\{ \begin{matrix} 1-Pe_{uf,0}^{-1}\tilde{x} & (i) \\ 1-Pe_{uf,0}^{-1}\tilde{x}\left( \alpha_{\min}+2-\tilde{x} \right) & (ii) \end{matrix} \right.$ (12)

$\tilde{u}_{d}\left( \tilde{x} \right)=\left\{ \begin{matrix} \alpha_{d/p}+\frac{h_{p}}{h_{d}}Pe_{uf,0}^{-1}\left( 1-\tilde{x} \right) & (i) \\ \alpha_{d/p}+\frac{h_{p}}{h_{d}}Pe_{uf,0}^{-1}\left( 1-\tilde{x} \right)\left[ \alpha_{\min}+\left( 1-\tilde{x} \right) \right] & (ii) \end{matrix} \right.$ (13)

which give us another dimensionless geometric ratio $h_{p}/h_{d}$.

*b. Flux expression*

The flux across the membrane has both a convective (ultrafiltration) and a diffusive component. For the latter, one would consider the boundary layer mass transfer coefficient on the blood and dialysate sides, $k_{p,i}$ and $k_{d,i}$, and the diffusive mass transfer coefficient across the membrane, $P_{m,i}$, also known as permeance. Because of the convection, one cannot use the resistance-in-series approach directly to determine the overall mass transfer (i.e. $N_{i}=P_{\mathrm{df},i}(c_{p,i}-c_{d,i})$, where $P_{\mathrm{df},i}=\left( k_{p,i}^{-1}+k_{d,i}^{-1}+P_{m,i}^{-1} \right)^{-1}$ is the overall mass transfer coefficient/permeance), though the approach should give a reasonable estimate at low ultrafiltration rates. Assuming pseudo-steady state across the membrane, one can derive a full expression for the flux that considers only the plasma and dialysate concentrations in the bulk for each species $i$, by solving the 1D convection-diffusion equation with steady convective flow in the fluid boundary layers and the membrane:^35,40^

$N_{i}=\frac{v_{\mathrm{uf}}S_{\infty,i}\left[ \exp\left( \mathrm{Pe}_{m,i}+\frac{v_{\mathrm{uf}}}{k_{p,i}}+\frac{v_{\mathrm{uf}}}{k_{d,i}} \right)c_{p,i}-c_{d,i} \right]}{\left( 1-S_{\infty,i} \right)\exp\left( \frac{v_{\mathrm{uf}}}{k_{d,i}} \right)\left[ e^{\mathrm{Pe}_{m,i}}-1 \right]+S_{\infty,i}\exp\left( \mathrm{Pe}_{m,i}+\frac{v_{\mathrm{uf}}}{k_{p,i}}+\frac{v_{\mathrm{uf}}}{k_{d,i}} \right)-S_{\infty,i}}$ (14)

where $S_{\infty,i}= (1 - \sigma_{i})$ is the sieving coefficient ($\sigma_{i}$ is the reflection coefficient), and $\mathrm{Pe}_{m,i}=S_{\infty,i}v_{\mathrm{uf}}/P_{m,i}$ is the dimensionless membrane Péclet number that compares ultrafiltration to diffusion across the membrane.

If diffusive mass transport is dominated by the membrane resistance rather than the boundary layer resistances, equation (14) reduces to:

$N_{i}=\frac{v_{\mathrm{uf}}S_{\infty,i}\left( e^{\mathrm{Pe}_{m,i}}c_{p,i}-c_{d,i} \right)}{e^{\mathrm{Pe}_{m,i}}-1}$ (15)

*Non-dimensionalization*

To non-dimensionalize, we set $\tilde{N}_{i}=N_{i}/(u_{p,\mathrm{in}}c_{0})$, which yields:

$\tilde{N}_{i}=\frac{h_{p}}{L}Pe_{\mathrm{uf}}^{-1}\frac{S_{\infty,i}\left[ \exp\left( \mathrm{Pe}_{m,i}+\frac{v_{\mathrm{uf}}}{k_{p,i}}+\frac{v_{\mathrm{uf}}}{k_{d,i}} \right)c_{p,i}-c_{d,i} \right]}{\left( 1-S_{\infty,i} \right)\exp\left( \frac{v_{\mathrm{uf}}}{k_{d,i}} \right)\left[ e^{\mathrm{Pe}_{m,i}}-1 \right]+S_{\infty,i}\exp\left( \mathrm{Pe}_{m,i}+\frac{v_{\mathrm{uf}}}{k_{p,i}}+\frac{v_{\mathrm{uf}}}{k_{d,i}} \right)-S_{\infty,i}}$ from (14) (16)

$\tilde{N}_{i}=\frac{h_{p}}{L}Pe_{\mathrm{uf}}^{-1}\frac{S_{\infty,i}\left( e^{\mathrm{Pe}_{m,i}}c_{p,i}-c_{d,i} \right)}{e^{\mathrm{Pe}_{m,i}}-1}$ from (15) (17)

where both ultrafiltration and membrane Péclet numbers are position-dependent in case ii:

$Pe_{\mathrm{uf}}^{-1}\left( \tilde{x} \right)=\frac{v_{\mathrm{uf}}\left( \tilde{x} \right)}{u_{p,\mathrm{in}}}\frac{L}{h_{p}}=\left\{ \begin{matrix} Pe_{uf,0}^{-1} & (i) \\ Pe_{uf,0}^{-1}\left[ \alpha_{\min} +2\left( 1-\tilde{x} \right) \right] & (ii) \end{matrix} \right.$ (18)

$Pe_{m,i}\left( \tilde{x} \right)=\left\{ \begin{matrix} Pe_{m,0,i} & (i) \\ Pe_{m,0,i}\left[ \alpha_{\min} +2\left( 1-\tilde{x} \right) \right] & (ii) \end{matrix} \right.$ (19)

where $\mathrm{Pe}_{m,0,i}=S_{\infty,i}v_{uf,0}/P_{m,i}$.

For clarity, we group the expression to the right of $Pe_{\mathrm{uf}}^{-1}$ into a function $g$:

$\tilde{N}_{i}\left( \tilde{x} \right)=\frac{h_{p}}{L}Pe_{\mathrm{uf}}^{-1} g\left( \tilde{x},c_{p,i}\left( \tilde{x} \right),c_{d,i}\left( \tilde{x} \right);S_{\infty,i},Pe_{m,0,i}, \alpha_{\min} \right)$ (20)

We proceed with the case where the boundary layer resistances are negligible (i.e. a single mass transfer coefficient/overall permeance $P_{\mathrm{df},i}$ can be used to model the diffusive mass transfer resistances in the solutions and across the membrane, i.e. $P_{\mathrm{df},i}\approx P_{m,i}$; we will use $P_{\mathrm{df},i}$ and $P_{m,i}$ interchangeably in the study) for $g$:^36^

$g\left( \tilde{x},c_{p,i}\left( \tilde{x} \right),c_{d,i}\left( \tilde{x} \right);S_{\infty,i},Pe_{m,0,i}, \alpha_{\min} \right)=\frac{S_{\infty,i}\left( e^{Pe_{m,i}}c_{p,i}-c_{d,i} \right)}{e^{Pe_{m,i}}-1}$ (21)

This expression can be further simplified in the following two cases. If $\mathrm{Pe}_{m,i}\ll1$, i.e. membrane mass transport is dominated by diffusion:

$\text{ }g\approx\frac{S_{\infty,i}\left( c_{p,i}-c_{d,i} \right)}{Pe_{m,i}}$ (22)

Plugging into the flux expression, one finds that the ultrafiltration rate terms drop out, and as expected, the concentration gradient across the membrane drives the toxin transport:

$\tilde{N}_{i}\left( \tilde{x} \right)=\frac{h_{p}}{L}Pe_{\mathrm{uf}}^{-1}g=\frac{h_{p}}{L}\frac{Pe_{uf,0}^{-1}}{Pe_{m,0,i}}S_{\infty,i}\left( c_{p,i}-c_{d,i} \right)=\frac{h_{p}}{L}\frac{P_{\mathrm{df},i}}{u_{p,\mathrm{in}}}\left( c_{p,i}-c_{d,i} \right)$ (23)

In contrast, if $\mathrm{Pe}_{m,i}\gg1$, the diffusive membrane permeance drops out and the mass transport does not depend on the dialysate concentration in the convection-dominant limit:

$g\approx S_{\infty,i}c_{p,i}$ (24)

Putting these all together, we can write:

$g_{i}=\left\{ \begin{matrix} \frac{S_{\infty,i}\left( c_{p,i}-c_{d,i} \right)}{Pe_{m,i}} & ; & Pe_{m,i}\ll1 \\ S_{\infty,i}c_{p,i} & ; & Pe_{m,i}\gg1 \\ \frac{S_{\infty,i}\left( e^{Pe_{m,i}}c_{p,i}-c_{d,i} \right)}{e^{Pe_{m,i}}-1} & ; & \mathrm{otherwise} \end{matrix} \right.$ (25)

Figure S3 illustrates the three different regimes and the expected concentration profiles across the membrane.


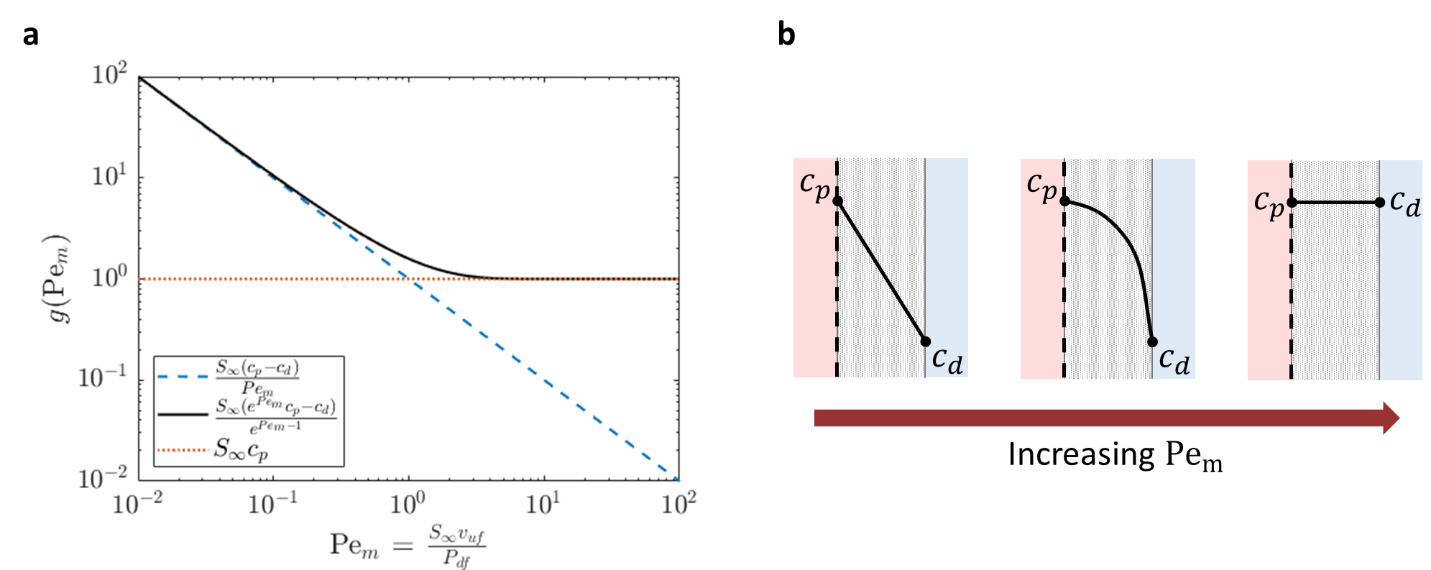


**Figure S3.** (a) $g$ as a function of $Pe_{m}$ for the demonstrative case of $c_{p}$ = 1, $c_{d}$ = 0, $S_{\infty}$ = 1.
(b) The corresponding (pseudo) steady state concentration profiles for the three regimes. From left to right: low (strong diffusion), intermediary, and high $Pe_{m}$ (strong convection/ultrafiltration).^35,36^

*c. Reaction kinetics*

The reaction term describes the interactions between the species. For simplicity, we consider five species: two free toxins, Z = A, B (*p*-cresyl sulfate pCS, indoxyl sulfate IS), the protein (albumin) which they bind to, P, and the bounded toxin-protein complexes, PZ = PA, PB. While we recognize the complexity of the binding kinetics of uremic toxins to albumin,^34,37,38^ here we follow the approach of previous literature and assume a simple 2^nd^-order association (forward) and 1^st^-order dissociation (backward) kinetics which should sufficiently capture the binding/unbinding events:^16,17,36^

$R_{j,i}=\left\{ \begin{matrix} -k_{Z}c_{j,Z}c_{j,P}+k_{-Z}c_{j,\mathrm{PZ}} & i=Z \\ -\sum_{Z} k_{Z}c_{j,Z}c_{j,P}+k_{-Z}c_{j,\mathrm{PZ}} & i=P \\ k_{Z}c_{j,Z}c_{j,P}-k_{-Z}c_{j,\mathrm{PZ}} & i=\mathrm{PZ} \end{matrix} \right.$ (26)

where the $k_{Z}$ [M^-1^ s^-1^] and $k_{-Z}$ [s^-1^] are the forward and backward rate constants for toxin Z. More complex kinetics and more toxins could be added or substituted into the equations as needed. Non-dimensionalization involves scaling all concentrations by the concentration scale $c_{0}$, and the volumetric reaction rate by the blood (plasma) channel convective time scale:

$\tilde{R}_{j,i}=\frac{L}{u_{p,\mathrm{in}}}\frac{R_{j,i}}{c_{0}}=\left\{ \begin{matrix} -\mathrm{Da}_{Z}c_{j,Z}c_{j,P}+\mathrm{Da}_{-Z}c_{j,\mathrm{PZ}} & i=Z \\ -\sum_{Z} \mathrm{Da}_{Z}c_{j,Z}c_{j,P}+\mathrm{Da}_{-Z}c_{j,\mathrm{PZ}} & i=P \\ \mathrm{Da}_{Z}c_{j,Z}c_{j,P}-\mathrm{Da}_{-Z}c_{j,\mathrm{PZ}} & i=\mathrm{PZ} \end{matrix} \right.$ (27)

Here again in equation (27), all concentrations are scaled concentrations (tilde neglected for clarity). Two Damköhler numbers per toxin emerge—a forward Damköhler number $\mathrm{Da}_{Z}=k_{Z} Lc_{0}/u_{p,\mathrm{in}}$ and a backward Damköhler number $\mathrm{Da}_{-Z}=k_{-Z} L/u_{p,\mathrm{in}}$. They compare the reaction rates with the blood (plasma) channel convection rate.

*d. Final non-dimensionalized expression*

Re-writing the governing equations (10-11) in terms of $g$, with the reaction term given by (27), we have:

$\frac{\partial c_{p,i}}{\partial\tilde{t}}=-\frac{\partial}{\partial\tilde{x}}\left[ {\tilde{u}_{p}c}_{p,i} \right]-Pe_{\mathrm{uf}}^{-1} g\left( \tilde{x},c_{p,i},c_{d,i};S_{\infty,i},Pe_{m,0,i}, \alpha_{\min} \right)+\tilde{R}_{p,i}$ (28)

$\frac{\partial c_{d,i}}{\partial\tilde{t}}=+\frac{\partial}{\partial\tilde{x}}\left[ {\tilde{u}_{d}c}_{d,i} \right]+\frac{h_{p}}{h_{d}}Pe_{\mathrm{uf}}^{-1} g\left( \tilde{x},c_{p,i},c_{d,i};S_{\infty,i},Pe_{m,0,i}, \alpha_{\min} \right)+\tilde{R}_{d,i}$ (29)

At steady state, the LHS is 0, and we can solve for the concentration profiles in the channel from these coupled ordinary differential equations given the boundary conditions (BCs) of inlet blood species concentration, $c_{p,i}(\tilde{x}=0)=c_{p,\mathrm{in},i}$, inlet dialysate species concentration, $c_{d,i}(\tilde{x}=1)=c_{d,\mathrm{in},i}$, and through the relation $c_{j,i}\left( \tilde{x} \right)={\tilde{u}_{j}c}_{j,i}/\tilde{u}_{j}\left( \tilde{x} \right)$ and the volumetric reaction terms.

Together, the governing equations for each species $i$ = A, B, P, PA, PB in blood or dialysate (28-29), along with the individual expressions for velocities $\tilde{u}_{p}$, $\tilde{u}_{d}$ (12-13), Péclet numbers $Pe_{\mathrm{uf}}$, $Pe_{m,i}$ (18-19), flux function $g$ (25), and kinetics (27) specify the device model. Adsorbents (e.g. albumin) could be introduced into the dialysate stream by changing the corresponding boundary condition at $\tilde{x}=1$.

The model derivation indicates that three non-species-specific (four if considering $\alpha_{\min}$ for case ii: linear ultrafiltration rate) and one species-specific ($Pe_{m,0,i}$) dimensionless parameter fully describes this system without binding reaction. Each toxin will introduce two Damköhler numbers (where the reaction rate constants are set by nature and are not design parameters).

*e. Equilibrium*

For kinetics rates significantly higher than mass transfer rates, the bulk species can be assumed to be in equilibrium, where the equilibrium constant (and its dimensionless form) is expressed as:

$K_{Z}=\frac{k_{Z}}{k_{-Z}}=\frac{c_{j,\mathrm{PZ}}}{c_{j,Z}c_{j,P}}\to\tilde{K}_{Z}=K_{Z}c_{0}=\frac{\mathrm{Da}_{Z}}{\mathrm{Da}_{-Z}}$ (30)

The fraction of toxin that is free, $\psi_{j,Z}$, is sometimes used to describe the equilibrium distribution. Its expression can be derived by rearranging the equilibrium equation:

$\psi_{j,Z}=\frac{c_{j,Z}}{{c_{j,Z}}_{\mathrm{tot}}}=\frac{c_{j,Z}}{c_{j,\mathrm{PZ}}+c_{j,Z}}=\frac{1}{1+ K_{Z}c_{j,P}}$ (31)

In the case where equilibrium is assumed, mass balance is only needed for 3 species (total A, B, albumin concentrations: $c_{j,A_{\mathrm{tot}}}$, $c_{j,B_{\mathrm{tot}}}$, $c_{j,P_{\mathrm{tot}}}$, where A and B are toxins), and the partitioning is determined by the equilibrium constants. Given $K_{A}$ and $K_{B}$, we can express the free albumin concentration, $c_{j,P}$, in relation to $c_{j,A_{\mathrm{tot}}}$, $c_{j,B_{\mathrm{tot}}}$, $c_{j,P_{\mathrm{tot}}}$ at any position along the channel:

$c_{j,P_{\mathrm{tot}}}=c_{j,P}\left( 1+\frac{K_{A}c_{j,A_{\mathrm{tot}}}}{1+ K_{A}c_{j,P}}+\frac{K_{B}c_{j,B_{\mathrm{tot}}}}{1+ K_{B}c_{j,P}} \right)= c_{j,P}\left( 1+\sum_{Z} \frac{K_{Z}c_{j,Z_{\mathrm{tot}}}}{1+ K_{Z}c_{j,P}} \right)$ (32)

The governing mass balances for the equilibrium case is the same as (28-29) except that the reaction term is omitted, and at each position, the cubic equation (32) is first solved for the albumin concentration, $c_{j,P}$, which is then used to determine the free toxin concentrations Z = A,B via:

$c_{j,Z}=\frac{{c_{j,Z}}_{tot}}{1+ K_{Z}c_{j,P}}$ (33)

$c_{j,Z}$ is then plugged into the flux expression $g\left( \tilde{x},c_{p,i=Z}\left( \tilde{x} \right),c_{d,i=Z}\left( \tilde{x} \right) \right)$ to determine the toxin flux across the membrane, which is mainly driven by the free components. The inclusion of more protein-bound toxins will require solving higher order polynomials numerically (32), and more sophisticated guessing for $c_{j,P}$ would be necessary to limit the computational time.

*f. Note on dimensionless numbers*

Alternative forms of dimensionless numbers can be derived by grouping and/or arranging the dimensionless numbers listed above, though the total number of dimensionless groups governing the system will not change.

For ease of comparison against the plasma-side channel convection rate, we define an inverse diffusive Péclet number that is the dimensionless mass transfer number (Table 1, main text):

$Pe_{df,i}^{-1}=S_{\infty,i}Pe_{uf,0}^{-1}Pe_{m,0,i}^{-1}=\frac{P_{df,i}}{u_{p,\mathrm{in}}}\frac{L}{h_{p}}\times\frac{w}{w}=\frac{P_{df,i} A_{m}}{(u_{p,\mathrm{in}}h_{p}w)}=\frac{K_{o}A_{i}}{Q_{p,\mathrm{in}}}$ (34)

We can also write:

$\tilde{Q}_{d/p}=\frac{Q_{d,\mathrm{in}}}{Q_{p,\mathrm{in}}}=\frac{u_{d,\mathrm{in}}}{u_{p,\mathrm{in}}}\frac{h_{d}}{h_{p}}\frac{w}{w}=\alpha_{d/p}\left( \frac{h_{p}}{h_{d}} \right)^{-1}$ (35)

The two Péclet numbers can also be defined using volumetric terms:

$Pe_{uf,0}^{-1}=\frac{v_{\mathrm{uf},0}}{u_{p,\mathrm{in}}}\frac{L}{h_{p}}\frac{w}{w}=\frac{Q_{\mathrm{uf},0}}{Q_{p,\mathrm{in}}}$ (36)

$Pe_{m,0,i}=\frac{S_{\infty,i}v_{uf,0}}{P_{m,i}}\frac{L}{L}\frac{w}{w}=\frac{S_{\infty,i}Q_{\mathrm{uf},0}}{K_{o}A}$ (37)

# S2. Simulation

Because of the stiffness of the equations, MATLAB’s fsolve and ode15s functions were used to implement the shooting method to solve the coupled ordinary differential equations for all species on both blood and dialysate sides. The shooting method involves starting at $\tilde{x}=0$, using the blood side inlet concentration BCs and guessing the dialysate outlet concentration $c_{d,i}(\tilde{x}=0)$, integrating forward until $\tilde{x}=1$, and using the dialysate inlet concentration $c_{d,i}(\tilde{x}=1)$ to adjust the initial guess until convergence.

# S3. Model assumptions and observations

Here we expand the discussion on the 1D formulation and lumped mass transfer coefficient, adsorption, and concentration assumptions made in the model.

*a. 1D assumption*

In our formulation, we lumped diffusive mass transport in the direction orthogonal to the membrane into an overall mass transfer coefficient (permeance) and wrote the governing equations only in the traverse ($x$) direction. We adopted this assumption since engineering features can always be added to promote mixing, and the various non-laminar flow profiles in different dialyzer designs made it necessary to adopt an average flow velocity.^23,24^ This formulation also neglects the reaction kinetics within the boundary layer (BL), which is a valid assumption if the BL is in near equilibrium (net reaction rates ≈ 0).

To verify the validity of this assumption, we performed a series of numerical simulations to examine the steady state diffusive mass transfer of a toxin/protein mixture in the direction orthogonal to the channel into the membrane (see Figure S4a). We solve for the case with no ultrafiltration, dialysate concentration of 0, and for various boundary layer thicknesses (10 nm – 10 μm) and membrane permeances (10^-6^ – 10^-4^ m s^-1^). From the concentration profiles, we calculate the reaction quotient $Q_{Z}=c_{\mathrm{PZ}}/(c_{P} c_{Z})$at the membrane and compare that with the equilibrium constant $K_{Z}$. We found that for all cases, equilibrium generally holds within the BL—there is only a maximum 14% difference between $Q_{Z}$ and $K_{Z}$ (for the extreme case of 10 μm BL and $P_{m}$ = 10^-4^ m s^-1^; see Table S1). Hence, even at a significantly high permeance, if the bulk is in equilibrium, then equilibrium generally holds across the boundary layer and at the membrane even as free toxins are removed (i.e. the perturbation is negligible compared to reaction kinetics). Thus, ignoring kinetics in the BL is reasonable, and binding/unbinding kinetics in the BL is only important when the BL resistance dominates (i.e. large BL thickness, high membrane permeance).

**Table S1.** Ratios of reaction quotient and equilibrium constant, $Q_{Z}/K_{Z}$, at the membrane surface.

| $\boldsymbol{P}\mathbf{m}$ **(m s^-1^)** | **pCS** | | | **IS** | | |
| --- | --- | --- | --- | --- | --- | --- |
| $\boldsymbol{\delta}_{\boldsymbol{p}}$ | **10^-6^** | **10^-5^** | **10^-4^** | **10^-6^** | **10^-5^** | **10^-4^** |
| **10 nm** | 1.0000 | 1.0001 | 1.0010 | 1.0000 | 1.0001 | 1.0010 |
| **100 nm** | 1.0001 | 1.0010 | 1.0100 | 1.0001 | 1.0010 | 1.0100 |
| **1 μm** | 1.0008 | 1.0084 | 1.0845 | 1.0008 | 1.0082 | 1.0824 |
| **10 μm** | 1.0013 | 1.0133 | 1.1368 | 1.0012 | 1.0123 | 1.1272 |

Furthermore, lumping diffusive mass transport into a single coefficient is analogous to using the bulk toxin concentration instead of the surface concentration to predict flux, i.e. saying Flux = $P_{m}\left( c_{1,p}-c_{1,d} \right)\approx P_{m}\left( c_{0,p}-c_{1+\delta_{d}/\delta_{p},d} \right)$, where $\delta_{p}$ and $\delta_{d}$ are the BL thicknesses, the first subscript denotes the position: 0 for the edge of the plasma BL, 1 for the membrane, and $1+\delta_{d}/\delta_{p}$ for the edge of the dialysate BL, and the second subscript denotes either the plasma ($p$) or dialysate (*d*) side. Our numerical model shows that there is negligible concentration drop across the BL for all permeance levels for BL thickness up to 1 μm. Table S2 summarizes the ratio of concentrations at the membrane to that at the bulk for different BL thicknesses and $P_{m}$, assuming dialysate side concentration is 0. For thicker BLs, the concentration drop can be adequately predicted with a resistance-in-series model (i.e. setting $P_{\mathrm{df}}^{-1}$ = $\left( D/\delta_{p} \right)^{-1}$+ $P_{m}^{-1}$ + $\left( D/\delta_{d} \right)^{-1}$, where $D$ is the diffusivity and $P_{m}$ is the membrane permeance of the toxin; Figure S4b), suggesting that (i) there is minimal reaction effect in the BL and (ii) when the ultrafiltration rate is relatively low (as in our case), a lumped permeance is adequate in predicting diffusive mass transfer behavior.

Nonetheless, the BL mass transfer resistance could have an effect on the overall mass transfer coefficient or permeance if the latter is high, and the dialyzer should be engineered to take that into consideration, otherwise further improvements in membrane permeance would not affect the overall mass transfer performance.^40^


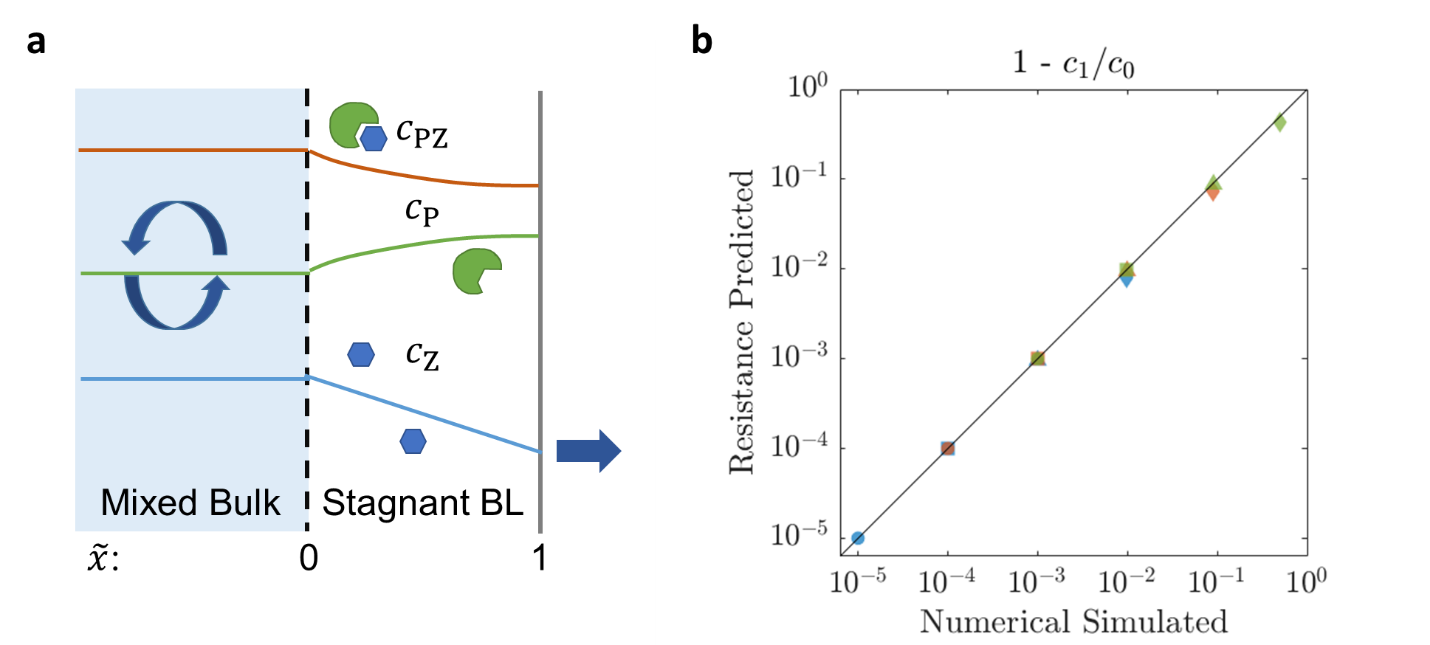


**Figure S4.** (a) Cartoon depiction of diffusive mass transfer across a stagnant boundary layer and the approximate concentration profiles. (b) Predicted (resistance-in-series) *vs*. numerically simulated 1 - concentration ratio at the membrane *vs*. in the bulk. Blue, orange, green refer to $P_{m}$ of 10^-6^, 10^-5^, 10^-4^ m s^-1^. Circle, square, triangle, diamond refer to BL thickness of 0.01, 0.1, 1, 10 μm. If the prediction matches the numerically simulated value, the points would lie on the 45° line, which is observed here, meaning the resistance-in-series model can predict most of the concentration changes across the BL, and reaction within the BL can be neglected. Only when permeance is high and BL is thick does $c_{1}$ differ substantially from $c_{0}$ (top right of graph).

**Table S2.** Ratios of free toxin concentration at the membrane surface (1) to the bulk (0), $c_{1}/c_{0}$.

| $\boldsymbol{P}\mathbf{m}$ **(m s^-1^)** | **pCS** | | | **IS** | | |
| --- | --- | --- | --- | --- | --- | --- |
| $\boldsymbol{\delta}_{\boldsymbol{p}}$ | **10^-6^** | **10^-5^** | **10^-4^** | **10^-6^** | **10^-5^** | **10^-4^** |
| **10 nm** | 1.000 | 0.9999 | 0.9990 | 1.000 | 0.9999 | 0.9990 |
| **100 nm** | 0.9999 | 0.9990 | 0.9901 | 0.9999 | 0.9990 | 0.9901 |
| **1 μm** | 0.9990 | 0.9904 | 0.9121 | 0.9990 | 0.9905 | 0.9123 |
| **10 μm** | 0.9921 | 0.9263 | 0.5666 | 0.9920 | 0.9259 | 0.5663 |

*Other mass transport assumptions and the introduction of adsorbents*

For simplicity and to focus on the role of permeance, we neglected species adsorption to the membrane surface as the adsorption capacity would depend on the membrane material. Furthermore, because of the small leakage of proteins, by first approximation we assumed no protein is transported across the membrane from the blood to dialysate, which is reasonable for practical low leakage membranes that should retain albumin in the blood stream. This simplifies the problem as the dialysate only has two species: pCS and IS. We also assumed $S_{\infty}$ = 1 for the free toxins, where typically value are ~0.99 – 1,^16,36^ but these differences are minimal as it is shown that the permeance is the main driver for membrane flux, not ultrafiltration. The governing equations can be solved using either the kinetics approach or assuming equilibrium.

However, we found that adding protein (adsorbent) to the dialysate introduces numerical instability to the kinetics problem, likely because of the high Damköhler numbers that result in a stiff problem. This is likely why quantitative adsorbent addition to dialysate, to our knowledge, has not been explored numerically through modeling, except for the infinite adsorption case which could be achieved by setting the dialysate toxin concentration to 0 to maximize diffusive flux (same as having an infinite dialysate flow rate), which is the maximum toxin removal achievable through dialysate changes.15 In contrast, we were able to overcome the problem using the equilibrium approach, and thus the adsorption simulations were performed using that.

*b. Concentrations*

The initial free toxin concentrations for pCS and IS were set based on the average values reported in the European Uremic Toxin Work group (EUTox) database.^9^ We had to adjust the total “accessible” albumin concentration, as some of the proteins would have their sites bound to other toxins and are thus not “accessible”. Because of the fast reaction rates, we assumed equilibrium holds between the free toxin, bounded-toxin, and the free accessible proteins, and is described by the equilibrium constants (Table 2, main text). We adopted free toxins concentration from the EuTox database: $c_{A},c_{B}=$ 2.6, 3.83 mg L^-1^ = 13.8, 18.0 μM, and set up functions to calculate the equilibrium free protein and protein-complexes concentrations via $c_{P}=c_{P_{\mathrm{tot}}}/(1+K_{A}c_{A}+K_{B}c_{B})$, $c_{\mathrm{PZ}}=K_{Z}c_{Z}c_{P_{\mathrm{tot}}}/(1+K_{A}c_{A}+K_{B}c_{B})$, $Z=A,B$. We then performed a least-square non-linear fit to determine the free protein concentration given the two equilibria and the free toxins concentrations which we matched, i.e., in order to find a consistent value for $c_{P_{\mathrm{tot}}}$, we minimized the 2-norm of the differences in protein-complex concentrations and their values given by EuTox ($c_{\mathrm{PA}},c_{\mathrm{PB}}=$ 20.4, 33.24 mg L^-1^ = 108, 156 μM) using the MATLAB function fmincon: $c_{P_{\mathrm{tot}}}=\mathrm{argmin} \left\| \left( c_{\mathrm{PA}}-{c_{\mathrm{PA}}}_{0},c_{\mathrm{PB}}-{c_{\mathrm{PB}}}_{0} \right) \right\|_{2}$. The bounded-toxin concentrations have to be re-adjusted to satisfy the equilibrium constraints for consistency, i.e. we used the $c_{P_{\mathrm{tot}}}$ value determined and the free toxin concentrations to calculate $c_{P}$, $c_{\mathrm{PA}}$, $c_{\mathrm{PB}}$ to be used as ODE boundary conditions. The resulting bounded-toxin concentrations~~, and~~ are within 8% and 4% of the EUTox values for pCS and IS respectively. The total “relevant” albumin concentration is then ~23 g L^-1^ as compared to the typical total albumin value of 35-45 g L^-1^.^2^ In this study, the initial concentrations used for free-pCS, bounded-pCS, free-IS, bounded-IS, and total albumin are: 13.8, 117.0, 18.0, 149.1, and 350.9 μM respectively.

*c. Parameter space selection*

There is no physical limit as to how high dialysate/blood flow ratio $\tilde{Q}_{d/p}$ can go by increasing $Q_{d,in}$, but for waste consideration we capped $\tilde{Q}_{d/p}$ at 20. In contrast, there is a maximum limit on the ultrafiltration rate capped by the amount of fluid in the plasma and interstitial body compartments (3.5, 12 L), as here in our model no additional fluid is introduced into the blood unlike in dilution hemodiafiltration. Hence, we set the upper limit of $Q_{\mathrm{uf}}$ to be 6 times the typical 10 mL min^-1^, i.e. removing a maximum of 15 L fluid over a 4 h dialysis period. Unlike small uremic solutes like urea or creatinine, limited numbers of experimental studies have been performed to measure the mass transfer coefficient, $K_{o}A$ = $P_{\mathrm{df}}A_{m}$, for PBUTs, and these were typically done *in vitro*. To match *in vivo* clearance measurements, *in vitro* values were recommended to be scaled down by 20%.^17,18^ Of the few measurements for IS or pCS’s $K_{o}A$, high flux polysulfone membranes yielded ~600 mL min^-1^ ($P_{\mathrm{df}}$ ~ 5×10^-6^ m s^-1^; similar permeance of 3×10^-6^ m s^-1^ was observed for a similar solute that contains an indole group, *L*-tryptophan, in diffusion cell studies), ^12,14,17–19,22^ while cellulose-based membranes (both low and high flux) yielded ~30 mL min^-1^ ($P_{\mathrm{df}}$ ~ 3×10^-7^ m s^-1^).^26^ Due to the uncertainty in PBUTs’ mass transfer coefficient values and their dependence on dialyzer/membrane types, we selected $P_{\mathrm{df}}$= 3 × 10^-6^ m s^-1^ to be a representative base value, though the range of values observed in literature were also covered in our analysis.

# S4. Kinetics versus equilibrium

Here we examined the validity of assuming equilibrium in the bulk fluid *vs.* using the more general kinetic expressions. Previous literature has used either cases but there has not been clear indication on when the equilibrium assumption is valid.^16,17,19,36^ To resolve this, we ran the simulations using both kinetics and equilibrium versions of the device model. Because of the large Damköhler numbers (≫1, i.e. reaction rates are extremely fast), we expected that the species at any position in the channel should be in near-equilibrium, unless there is significant perturbation pulling the system away from equilibrium, e.g. at high membrane fluxes.

We demonstrated that both approaches yield the same toxin removal for all cases examined (Figure S5), since even at permeances of 10^-4^ m s^-1^ the reaction is still significantly faster than mass transport, as indicated by the comparison of reaction kinetics *vs.* permeance through combining relevant dimensionless groups: $\mathrm{Da}_{i}Pe_{df,i}^{-1}$ $=k_{Z}c_{0}h_{p}S_{\infty,i}/P_{df,i}=306\gg1$. This suggests that, for reasonable permeances, the protein binding equilibrium cannot be easily broken unless chemical potentials are shifted, e.g. by introducing a competitive binder.^15,16^ Furthermore, this illustrates that whether kinetics or equilibrium were used in simulation would not yield substantially different results. We proceeded our analysis with the more general and faster kinetics approach except for the case of albumin adsorbent introduction into the dialysate, which was more readily solvable using equilibrium.


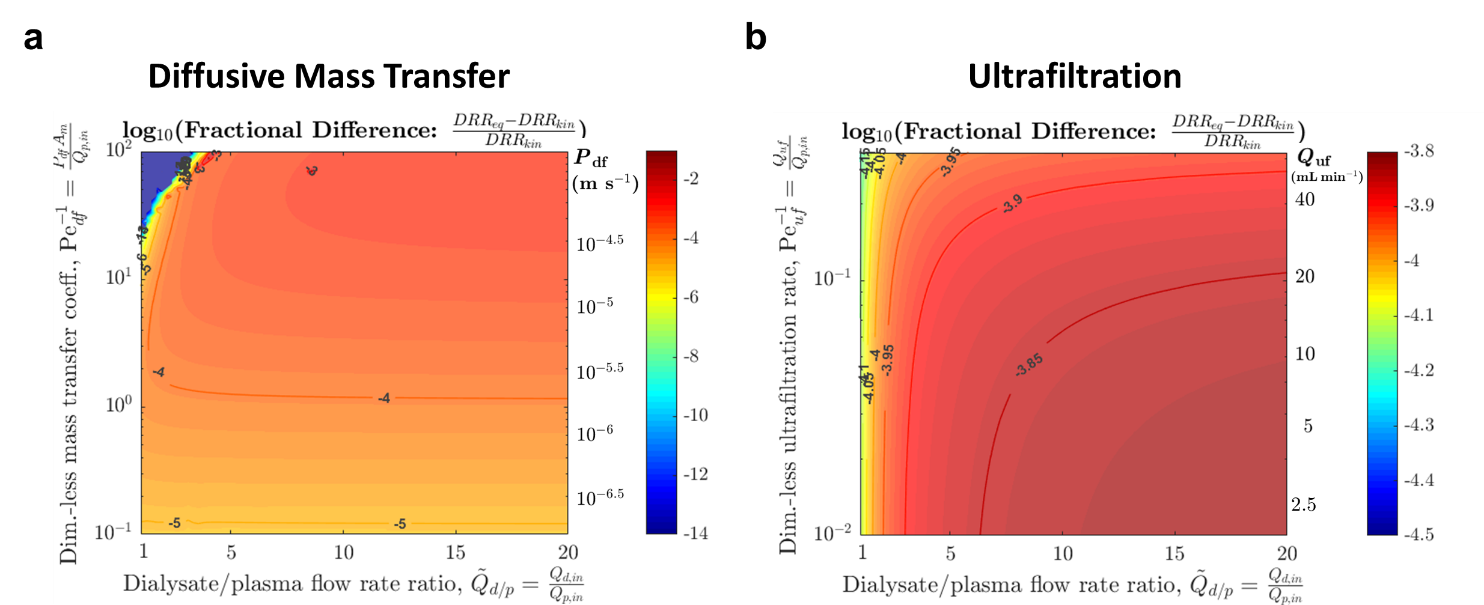


**Figure S5.** Fractional difference in device removal ratio (DRR) values for indoxyl sulfate between equilibrium and kinetics models, i.e. (equilibrium - kinetics)/kinetics, for varying dialysate/plasma flow rate ratio and (a) diffusive mass transfer coefficient/permeance $\mathrm{Pe}_{\mathrm{df}}^{-1}\propto P_{\mathrm{df}}$ or (b) ultrafiltration rate $\mathrm{Pe}_{\mathrm{uf}}^{-1}\propto$ $Q_{\mathrm{uf}}$, while holding other parameters constant as in Figure 2a. The contour levels and color are in log scale. Most of the values were well within 1%, other than some numerical instability encountered at high permeance levels and low dialysate flow rates.

# S5. Compartment model specification

We followed Maheshwari et al.’s (2019) formulation to describe the toxin partitioning in the human body using three compartments (intracellular = *ic*, interstitial = *is*, plasma = *pl*).^16^ Toxins are generated in the intracellular compartment, where no protein (albumin) is present, and are transported into the interstitial at a rate defined by the mass transfer coefficient $k_{ic}$ = 100 mL min^-1^. The interstitial compartment then exchanges toxins (but not proteins) with the plasma through non-convective mass transfer $k_{is}$ = 1135 mL min^-1^ and convective mass transfer via fluid removal due to ultrafiltration $Q_{\mathrm{uf}}$ = 10 mL min^-1^, resulting in a change in fluid volume. The plasma exchanges both toxins and proteins with the hemodialysis device. The initial compartment volumes are: $V_{ic}$ = 28 L (constant), $V_{is,0}$ = 12 L, and $V_{pl,0}$ = 3.5 L.^16^

Mass balance in the intracellular (ic) for toxins $i$ = A (pCS), B (IS) yields:

$V_{ic}\frac{dc_{ic,i}}{dt}=G_{i}-k_{ic,i}\left( c_{ic,i}-c_{is,i} \right)$ $i=A,B$ (38)

where the generation rates are $G_{A}$ = 0.02557 mg min^-1^ and $G_{B}$ = 0.02477 mg min^-1^.^16^

For the interstitial (is), differential mass balance is only needed for A, B, PA, PB, since the total protein mass, $c_{is,P_{\mathrm{tot}}}$, is constant:

$\frac{d\left( V_{is}c_{is,i} \right)}{dt}=R_{i}V_{is}+\left\{ \begin{matrix} k_{ic,i}\left( c_{ic,i}-c_{is,i} \right)-k_{is,i}\left( c_{is,i}-c_{p,i} \right)\text{ }-\frac{Q_{\mathrm{uf}}}{V_{pl}+V_{is}} (V_{is}c_{is,i})\text{ } & i=A,B \\ 0 & i=PA,PB \end{matrix} \right.$ (39)

$V_{is}c_{is,P}=V_{is}\left( c_{is,P_{\mathrm{tot}}}-c_{is,PA}-c_{is,PB} \right)$ (40)

For the plasma (pl), differential mass balance is performed for all 5 species, and the fluid is pumped through the dialyzer and back into the plasma compartment:

$\frac{d\left( V_{pl}c_{pl,i} \right)}{dt}=R_{i}V_{pl}-Q_{p}c_{pl,i}+\left( Q_{p}-Q_{\mathrm{uf}} \right)c_{p,x=L (out),i}$

$+\left\{ \begin{matrix} k_{is,i}\left( c_{is,i}-c_{p,i} \right) +\frac{Q_{\mathrm{uf}}}{V_{pl}+V_{is}} (V_{is}c_{is,i}) & i=A,B \\ 0 & i=P,PA,PB \end{matrix} \right.$ (41)

Fluid balance in the interstitial and plasma compartments gives:

$\frac{dV_{is}}{dt}=-\frac{V_{is}}{V_{pl}+V_{is}}Q_{\mathrm{uf}}$ (42)

$\frac{dV_{pl}}{dt}=-\frac{V_{pl}}{V_{pl}+V_{is}}Q_{\mathrm{uf}}$ (43)

where the rate of volume removal from a compartment is assumed to be proportional to the fractional volume of that compartment multiplied by the ultrafiltration rate.

The volume equations can be solved directly using separations of variable after adding (42-43):

$\left[ \frac{dV_{is}}{dt}=-\frac{V_{is}}{V_{pl}+V_{is}}Q_{\mathrm{uf}} \right]$ + $\left[ \frac{dV_{pl}}{dt}=-\frac{V_{pl}}{V_{pl}+V_{is}}Q_{\mathrm{uf}} \right]$ $\Rightarrow$ $\frac{d{(V}_{pl}+V_{is})}{dt}=-Q_{\mathrm{uf}}$ $\Rightarrow V_{pl}+V_{is}=V_{pl,0}+V_{is,0}-Q_{\mathrm{uf}} t$

$\frac{dV_{pl}}{dt}=-\frac{V_{pl}}{V_{pl,0}+V_{is,0}-Q_{\mathrm{uf}}t\text{ }}Q_{\mathrm{uf}}$ $\Rightarrow$ $\int_{V_{pl,0}}^{V_{pl}} \frac{1}{\hat{V}_{pl}'}d\hat{V}_{pl}=-Q_{\mathrm{uf}}\int_{0}^{t} \frac{1}{V_{p,0}+V_{is,0}-Q_{\mathrm{uf}}\hat{t}\text{ }}d\hat{t}$

$V_{pl}=V_{pl,0}\left( 1-\frac{Q_{\mathrm{uf}}}{V_{pl,0}+V_{is,0}}t \right)$ (44)

$V_{is}=V_{is,0}\left( 1-\frac{Q_{\mathrm{uf}}}{V_{pl,0}+V_{is,0}}t \right)$ (45)

To non-dimensionalize, we scale time by the blood convective time scale $t_{\mathrm{scale}} = L/u_{p.\mathrm{in}}$, volume by the sum of interstitial and plasma volumes $V_{pl,0}+V_{is,0}$, and concentration by the initial total toxin concentration in the intracellular compartment $c_{ic,A,0}+c_{ic,B,0}$. Again, the tilde denotes dimensionless parameters while $c$ is kept for dimensionless concentrations for clarity.

$\frac{d({\tilde{V}_{ic}c}_{ic,i})}{d\tilde{t}}=\tilde{G}_{i}-\tilde{k}_{ic,i}\left( c_{ic,i}-c_{is,i} \right)\text{ }$ $i=A,B$ (46)

$\frac{d\left( \tilde{V}_{is}c_{is,i} \right)}{d\tilde{t}}=\tilde{R}_{is,i}\tilde{V}_{is}\text{ }$

$+\left\{ \begin{matrix} \tilde{k}_{ic,i}\left( c_{ic,i}-c_{is,i} \right)-\tilde{k}_{is,i}\left( c_{is,i}-c_{pl,i} \right)\text{ }-\tilde{Q}_{\mathrm{uf}}\tilde{V}_{is,0} c_{is,i}\text{ } & i=A,B \\ 0 & i=PA,PB \end{matrix} \right.$ (47)

$\tilde{V}_{is}c_{is,P}=\left[ \tilde{V}_{is}c_{is,P_{tot}} \right]_{0}-\tilde{V}_{is}c_{is,PA}-\tilde{V}_{is}c_{is,PB}$ (48)

$\frac{d\left( \tilde{V}_{pl}c_{pl,i} \right)}{d\tilde{t}}=\tilde{R}_{pl,i}\tilde{V}_{pl}-\tilde{Q}_{p}c_{pl,i}+(\tilde{Q}_{p}-\tilde{Q}_{\mathrm{uf}})c_{p,\tilde{x}=1,i}$

$+\left\{ \begin{matrix} \tilde{k}_{is,i}\left( c_{is,i}-c_{pl,i} \right) +\tilde{Q}_{\mathrm{uf}}\tilde{V}_{is,0}c_{is,i} & i=A,B \\ 0 & i=P,PA,PB \end{matrix} \right.$ (49)

The method of lines is used to solve the partial differential equations in MATLAB, with finite differencing in space and a total of 10$n$ + 11 variables. $n$ is the number of discretization in the device and the factor of 10 is due to the 5 species in the 2 bulk compartments. $n$ = 101 is sufficient to model the mass transfer in the device, and increasing $n$ does not yield different results. The time resolution is below 0.1 min for the ODEs.


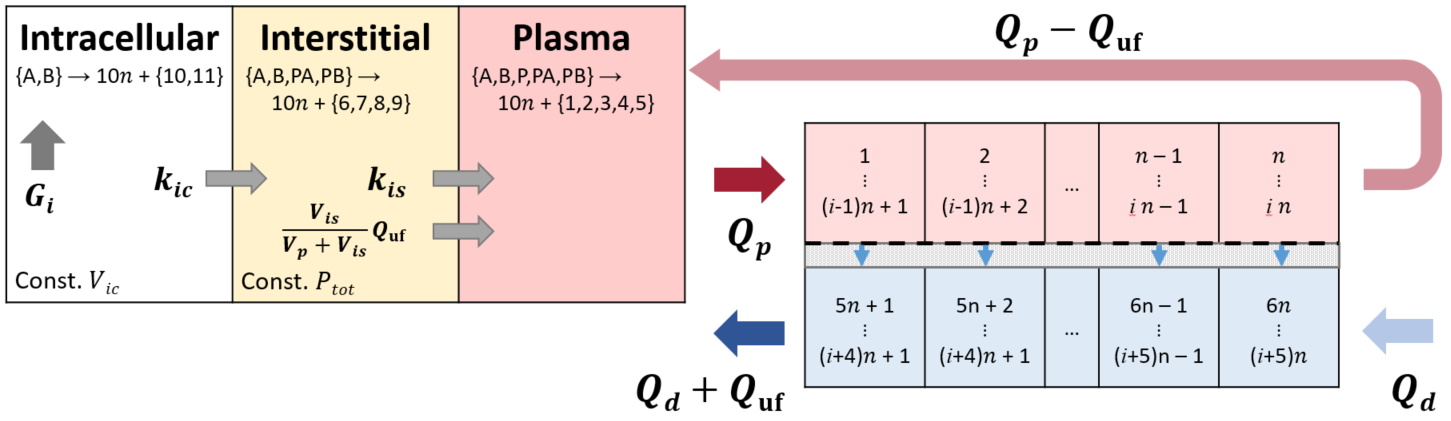


**Figure S6.** Compartment model solved using the method of lines. The body is divided into 3 compartments and connected to the device, which is discretized into 10$n$ cells, with $i$ = 1, …, 5 as the chemical species.

Note that it takes $L/u_{p,\mathrm{in}}$ ~30 s to fill the dialyzer up at a rate of $Q_{p}$. For numerical simplicity, we assumed that the dialyzer was initially filled with dialysate with no toxin, which results in a slightly higher toxin removal from the plasma compartment for ~30s. Alternatively, one could imagine initializing the numerical system such that the dialyzer plasma channel is initially filled with dialysate in equilibrium with the plasma. This would avoid the dilution effect but the volume and toxin mass have to be subtracted from the plasma compartment to keep the same initial mass.

# S6. Comparison between pCS and IS results

pCS and IS give almost identical results since their reaction parameters are very similar (Table 2, main text). A comparison of the two toxins’ DRRs for different parameter sweeps for the kinetic device model is included below.


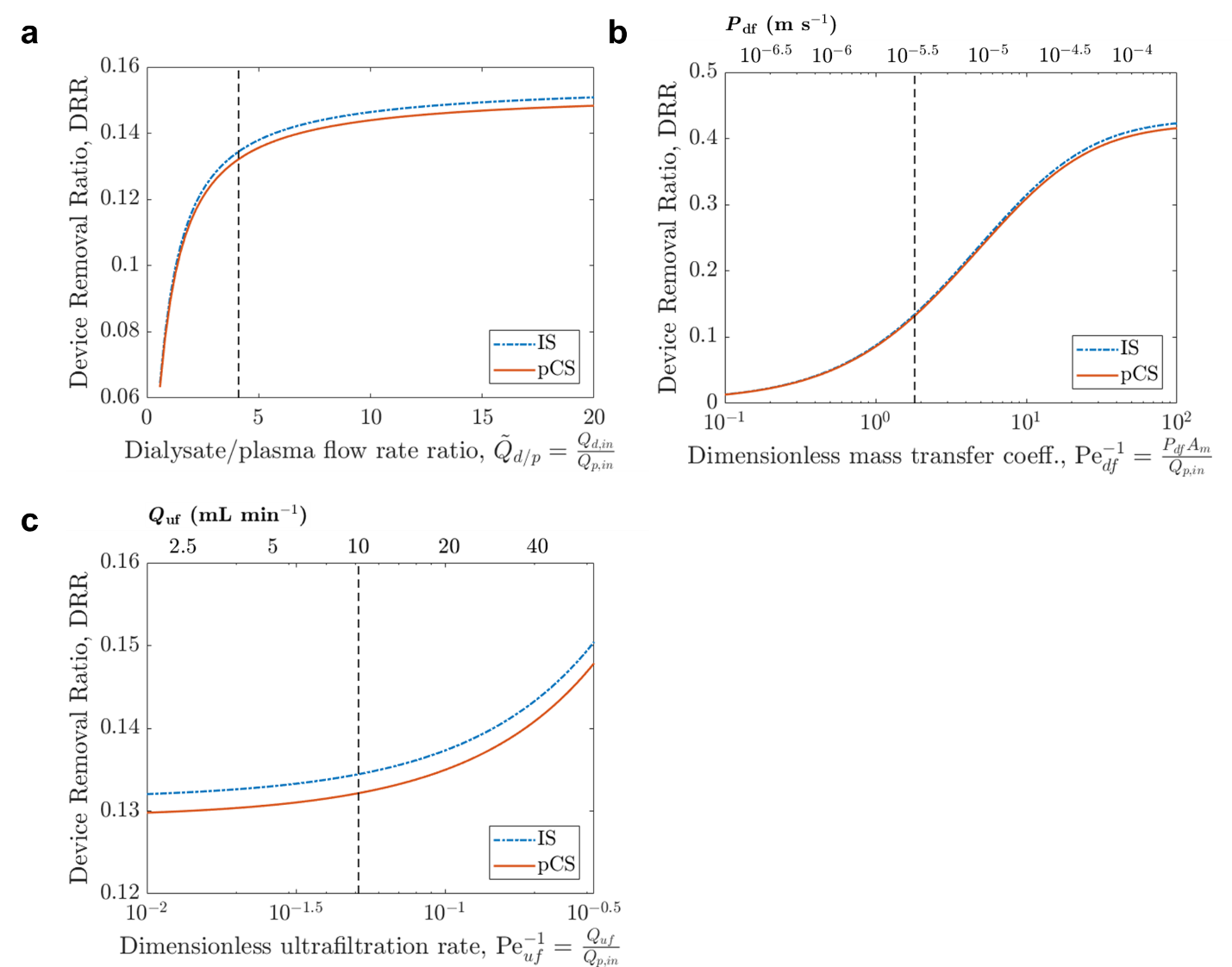


**Figure S7.** Device model: Effect of changing (a) dialysate/plasma flow rate ratio $\tilde{Q}_{d/p}$, (b) dimensionless diffusive mass transfer coefficient $\mathrm{Pe}_{\mathrm{df}}^{-1}\propto P_{\mathrm{df}}$, and (c) dimensionless ultrafiltration rate $\mathrm{Pe}_{\mathrm{uf}}^{-1}\propto Q_{\mathrm{uf}}$ on the device removal ratio DRR for IS (blue dot-dash) and pCS (red line). Note the difference in $y$ scale across the plots.

# S7. Removal metrics

*a. Device model*

*Device Removal Ratio (DRR)*

As mentioned in the main text, the “device removal ratio” for toxin $Z$ can be used to describe the toxin removal performance in a dialyzer. We can write DRR in terms of dimensionless numbers:

$\mathrm{DR}R_{Z}=\frac{h_{d}}{h_{p}}\frac{u_{d}\left( \tilde{x}=0 \right)}{u_{p,\mathrm{in}}}\frac{c_{d,Z_{\mathrm{tot}}}\left( \tilde{x}=0 \right)}{c_{p,Z_{\mathrm{tot}}}\left( \tilde{x}=0 \right)}=\left( \frac{h_{p}}{h_{d}} \right)^{-1}\left( \alpha_{d/p}+Pe_{uf,0} \right)\frac{c_{d,Z_{\mathrm{tot}}}\left( \tilde{x}=0 \right)}{c_{p,Z_{\mathrm{tot}}}\left( \tilde{x}=0 \right)}$

$=1-\frac{u_{p}\left( \tilde{x}=1 \right)}{u_{p,in}}\frac{c_{p,Z_{\mathrm{tot}}}\left( \tilde{x}=1 \right)}{c_{p,Z_{\mathrm{tot}}}\left( \tilde{x}=0 \right)}=1-(1-Pe_{uf,0})\frac{c_{p,Z_{\mathrm{tot}}}\left( \tilde{x}=1 \right)}{c_{p,Z_{\mathrm{tot}}}\left( \tilde{x}=0 \right)}$ (50)

*Clearance*

Another commonly used metric is the dialyzer clearance in mL min^-1^. This metric, to the best of our knowledge, was first applied to describe urea removal by dialysis in 1985, and can be defined as the volume of plasma that would be completely cleared of a toxin per unit time of dialysis. Operationally, a definition used in literature that does not require the consideration of body compartments is:^19,36^

$K_{Cl,\dot{M}}=$ $\frac{total toxin transport crossing the membrane [mol \min^{-1} ]}{\mathrm{plasma}\left( \mathrm{inlet} \right) solute concentration [mol \mathrm{mL}^{-1}]}=\frac{\dot{M}_{Z_{\mathrm{tot}}}}{c_{p,Z_{\mathrm{tot}}}\left( \tilde{x}=0 \right)}$ (51)

This gives the performance of the dialyzer at any snap-shot in time, as the membrane flux will vary across time during the dialysis period. At steady state (SS), mass balance means that $\dot{M}_{Z_{\mathrm{tot}},SS}=\left. \left( Q_{d}c_{d,Z_{\mathrm{tot}}} \right) \right|_{\tilde{x}=0}$, i.e. whatever crosses the membrane has to be removed from the dialysis channel outlet. Thus, for a device operating in steady state, the DRR is related to clearance by $\mathrm{DR}R_{Z}=K_{Cl,\dot{M},SS}/Q_{p,\mathrm{in}}$.

Another clearance expression that Meyer et al. (2004) used and Maheshwari et al. (2017) reproduced in their work is:^17,19^

$K_{Cl,K_{o}A}=Q_{p}\left( 1-\frac{\psi-\theta}{\phi\psi-\theta} \right)$ , $\phi=\exp\left( K_{o}A\left( \frac{\psi}{Q_{p}}-\frac{1}{Q_{d}} \right) \right)$ , $\theta=\frac{Q_{p}}{Q_{d}}$ (52)

where $\psi$ is the free fraction of toxins, defined in equation (31). This equation is typically used to calculate $K_{o}A$ of the membrane dialyzer. However, it is important to note that (a) this expression is derived from an analytical solution to the differential equations which describe diffusive transport and does not consider ultrafiltration,^33,36^ (b) it assumes that $\psi$ is constant along the length of the dialyzer, which is not necessarily true, especially at high permeance, and (c) in an actual system connected to the body, this clearance value will vary across time as toxins are removed from the body which changes $\psi$ (Figure S8). Given these drawbacks, DRR and $K_{Cl,\dot{M}}$ are preferred over $K_{Cl,K_{o}A}$ as a metric to describe the actual clearance of the system, though $K_{Cl,K_{o}A}$ can be calculated more easily without the need to know channel outlet concentrations.


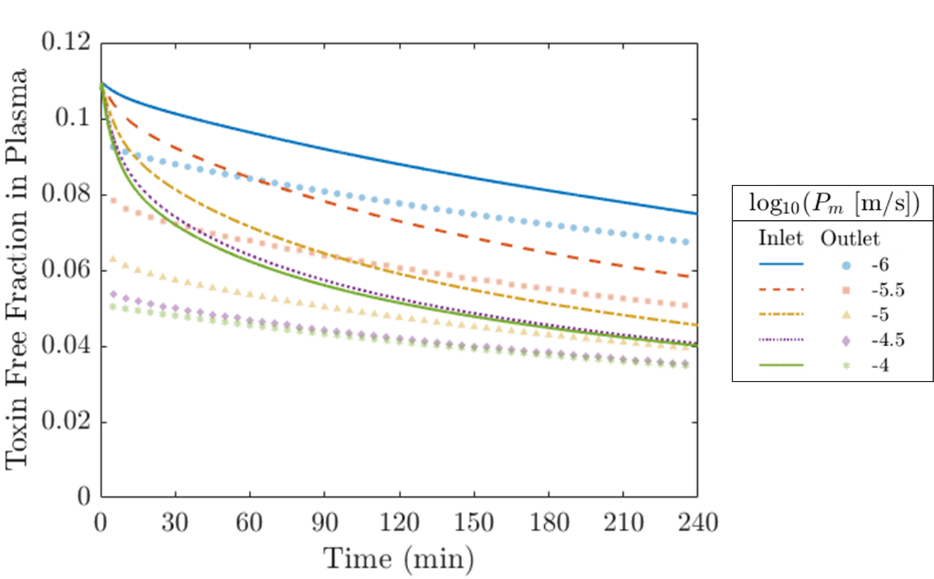


**Figure S8.** Variation of the free fraction of toxin in the plasma over time in the plasma channel inlet (lines) and outlet (dots) of the dialyzer for various permeance levels in the compartment model with base values. The vertical difference between two curves of the same color denotes the free fraction difference at the inlet and outlet, which is not constant across time. This difference is larger for high permeances.

*b. Compartment model*

*Clearance*

Another clearance expression commonly used for non-PBUTs but sometimes for PBUTs is:^14,17^

$K_{Cl,1}=\frac{1}{t}\frac{\Delta q_{\mathrm{net}}}{\left( \frac{c_{pl}\left( t=0 \right)-c_{pl}\left( t \right)}{\ln c_{pl}\left( t=0 \right)-\ln c_{pl}\left( t \right)} \right)}$ (53)

where $\Delta q_{\mathrm{net}}$ is the net (mass/molar) removal defined in main text equation (10). This expression is used to measure the dialysis dose and is derived from the commonly-used *single-compartment* solute clearance model for simple toxins.^21^ In that model, the compartment (distribution) volume $V$ is approximated as constant, and the mass balance can be solved via separations of variables:^10^

$\frac{dq}{dt}=\frac{d\left( Vc_{pl} \right)}{dt}=V\frac{dc_{pl}}{dt}=-K_{Cl,1}c_{pl}$

$\frac{dc_{pl}}{c_{pl}}=-\frac{K_{Cl,1}}{V}dt$ $\ln c_{pl}\left( t=0 \right)-\ln c_{pl}\left( t \right)=\frac{K_{Cl,1}}{V}t$ (54)

For negligible volume change in a 1-compartment model, net removal is $\Delta q=V\Delta c$. Substituting $V=\Delta q/\Delta c$ into (54) and rearranging yields the clearance expression in (53). However, $K_{Cl,1}$ explicitly assumes a 1-compartment model that neglects the toxin partitioning within different body compartments, and thus it should be interpreted with caution for PBUTs.

The three clearance metrics (51-53) should be the same *if* there is no protein binding, only 1 compartment, and diffusive transport dominates over convective (ultrafiltration) transport. However, this is not the case for PBUTs, and Figure S9 illustrates how they relate to each other.

*Reduction Ratio*

The reduction ratio, RR, is frequently used to estimate the amount of toxin removed from the blood/plasma. It is defined as:^16,17^

RR = $1-\frac{c_{pl}(t)}{c_{pl}(t=0)}$ (55)

where the concentrations refer to the total toxin (free + bounded). RR is a simpler metric because only concentrations are needed for its calculation which could be readily measured. However, in some cases RR can be problematic—it can go negative even as toxins are removed. This occurs when permeance is low and ultrafiltration reduces the plasma volume substantially, which raises toxin concentration. This can be shown through a differential mass balance for a compartment:

$\frac{dq}{dt}=\frac{d(Vc)}{dt}=c\frac{dV}{dt}+V\frac{dc}{dt}$ $\Rightarrow$ $\frac{dc}{dt}=\frac{1}{V}\frac{dq}{dt}-\frac{c}{V}\frac{dV}{dt}$ (56)

Even if mass is decreasing ($dq/dt<0$), the concentration could be increasing because of the concentrating effect as the volume is decreasing due to ultrafiltration ($dV/dt<0$). Hence, we define a more physically meaningful RR–the mass reduction ratio, MRR–which considers the mass instead of the concentration, such that when toxins are removed MRR is strictly positive:

$MRR= 1-\frac{q_{pl}(t)}{q_{pl}(t=0)}= 1-\frac{V_{pl}(t)c_{pl}(t)}{V_{pl}(t=0)c_{pl}(t=0)}$ (57)

This will overcome the dilution problem that RR faces, although changes in the plasma volume needed to be estimated, e.g. by using the ultrafiltration rate and equation (44).

*Comments*

The single-compartment clearance ($K_{Cl,1}$) and removal ratios (RR and MRR), though telling, might not be the most appropriate metrics to describe a multi-compartment system with protein-binding/toxin partitioning because (a) they only involve plasma concentrations and not the other compartments, and it takes time for concentration changes in the plasma to translate to changes in the interstitial and intracellular compartments, and (b) the fluid volume in the body changes due to ultrafiltration. If the clinically relevant level is the plasma concentration then these metrics are fine. However, if the interstitial and intracellular toxin levels are also important, then the (fractional) net removal, ($f_{\Delta q_{\mathrm{net}}}$) $\Delta q_{\mathrm{net}}$, defined in the main text, might be more appropriate. As discussed above, mass changes are more representative than concentration changes in depicting removal performance, but the former require estimating the distribution volumes which are likely to vary across different patients and might be hard to predict.

Net removal can be determined easily in simulations by looking at the changes in concentrations and volumes in all compartments, whereas in practice only the plasma and dialysate content can be readily measured. Thus, to measure net removal, one would have to measure the concentration of the toxin in the dialysate over time and multiply it by the dialysate outlet flow rate and integrate across the dialysis duration, which could become possible with advances in inline measurements; alternatively, the net removal can be obtained more easily by measuring the toxin concentration in the dialysate that is collected over the duration of the dialysis session (and mixed well) and multiplying it by the dialysate volume. Since clearance and RRs involve physically measurable quantities relevant to the patient (plasma concentration), they are more operationally feasible and are thus used more in literature.

Figure S9 shows how these metrics change over a dialysis session. RRs are lower than MRRs due to the concentrating effect of ultrafiltration (i-iv). The free toxins are more easily removed than protein-bounded ones, so free RRs and MRRs are significantly higher than their total counterparts (iii-iv *vs.* i-ii). RR and MRR overestimates the actual of amount of toxin removed *vs.* fractional net removal because the former only considers changes in the plasma compartment, where toxins are easier to remove as they are pumped directly into the dialyzer (i-iv *vs.* v). Concentrations in the plasma are lower than in the interstitial compartment, and the differences are larger at higher rates of removal (vii *vs.* viii). All clearance metrics qualitatively follow the same trends (ix-xi), with $K_{Cl,K_{o}A}$ and $K_{Cl,\dot{M}}$ deviating less from each other especially at lower permeances, since the free fraction differences between inlet and outlet are still relatively small. DRR matches the shape of $K_{Cl,\dot{M}}$ since the latter can be recovered from the former via multiplying by $Q_{p}$. While all literature studies only report a single clearance value (using at the final dialysis time),^5,13,16,17,19,36^ here we demonstrated that all clearance metrics vary across time. Hence, it would be very useful for studies to mention how clearance values are calculated. The results also show that the commonly used clearance metrics have limitations and should be interpreted carefully.


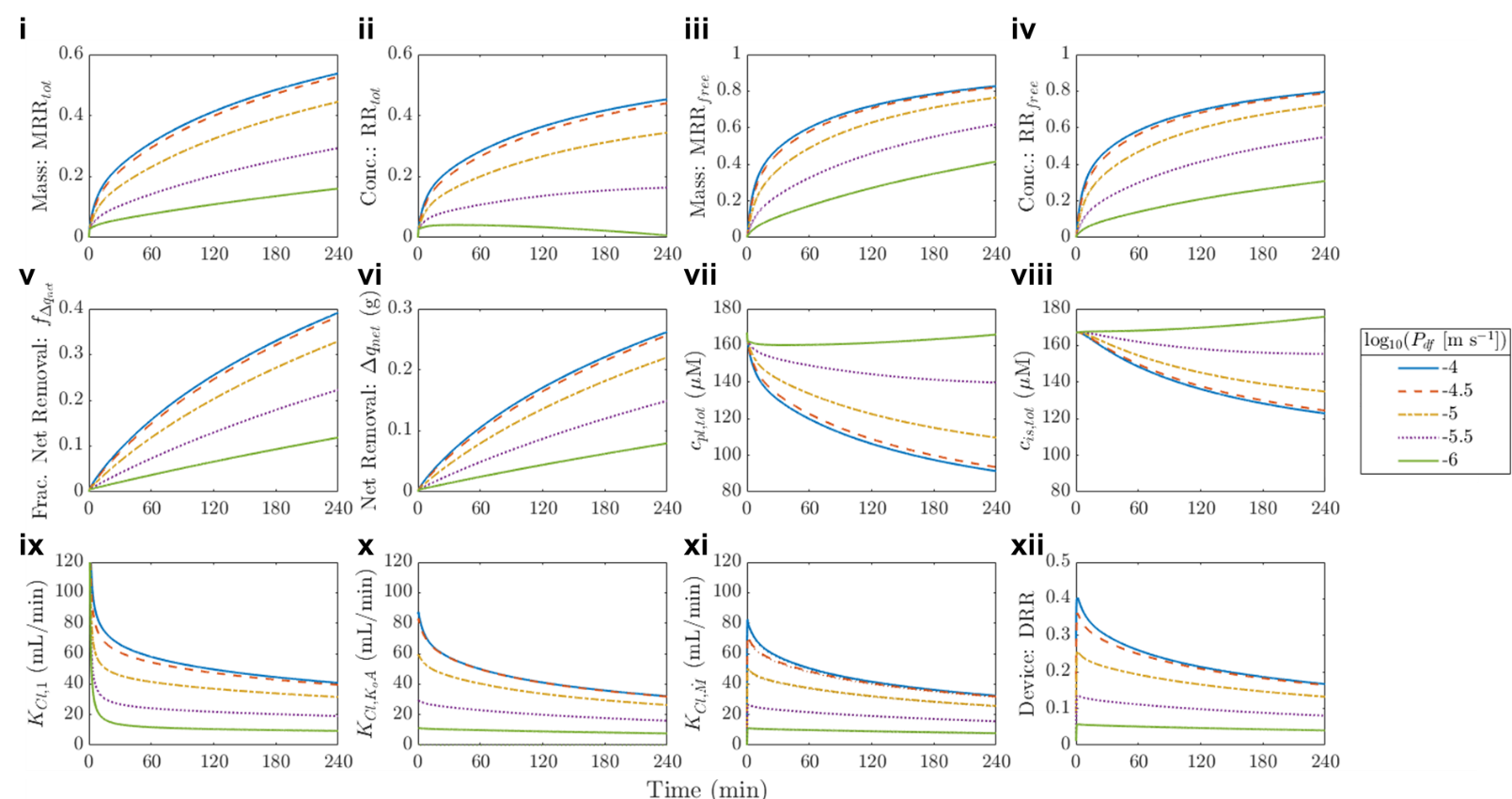


**Figure S9.** Changes in IS removal metrics over a 4 h dialysis period for various permeances. Top row: Mass reduction ratios (MRR) and RR for (i-ii) total toxins and (iii-iv) only free toxins. Middle row: net removal as (v) fraction and (vi) mass; total toxin concentrations in (vii) plasma and (viii) interstitial. Bottom row: clearances (ix) single-compartment $K_{Cl,1}$, (x) free fraction (plasma chamber) $K_{Cl,K_{o}A}$, (xi) membrane mass transport $K_{Cl,\dot{M}}$; (xii) device removal ratio DRR.

Our results highlight the need to converge on metric(s) that best describe PBUT removal performance to facilitate cross-study comparison—whether to continue using existing metrics but with clear caveats on their limitations, or to establish more suitable metrics. Furthermore, we argue that having a toxin removal metric for *the device itself* (e.g. DRR) that relates to flow and membrane characteristics can inform better module and membrane design.

# S8. Urea compartment model

A two-compartment model was adopted from literature to describe the effect of changing operation parameters on urea removal. In literature, both regional blood flow and serial two-compartment models have been used to describe urea kinetics.^8^ For simplicity and benchmarking purpose, we adapted the PBUT compartment model for urea, adjusting the toxin generation rate to 5 mg min^-1^, initial urea concentration to 1.5 g L^-1^ (equivalent to urea nitrogen of 68 mg dL^-1^), and using only intracellular and extracellular compartments with distribution volumes of 26.7 and 13.3 L (total 40 L) respectively (i.e. merging “plasma” and “interstitial” because of the fast transport between them due to urea’s small size; $k_{ic}$ = 600 mL min^-1^; see Figure S10).^3,27^ The model also neglects the presence of the blood circulation through the heart, and assumes direct removal of volume from the intracellular compartment and no residual renal clearance.^8,25^ Note that the above numbers can vary quite substantially across different patients.^28,32^ We emphasize that this model is only an estimate for urea clearance performance. More accurate predictions would require a more sophisticated model calibrated with patient characteristics and data.


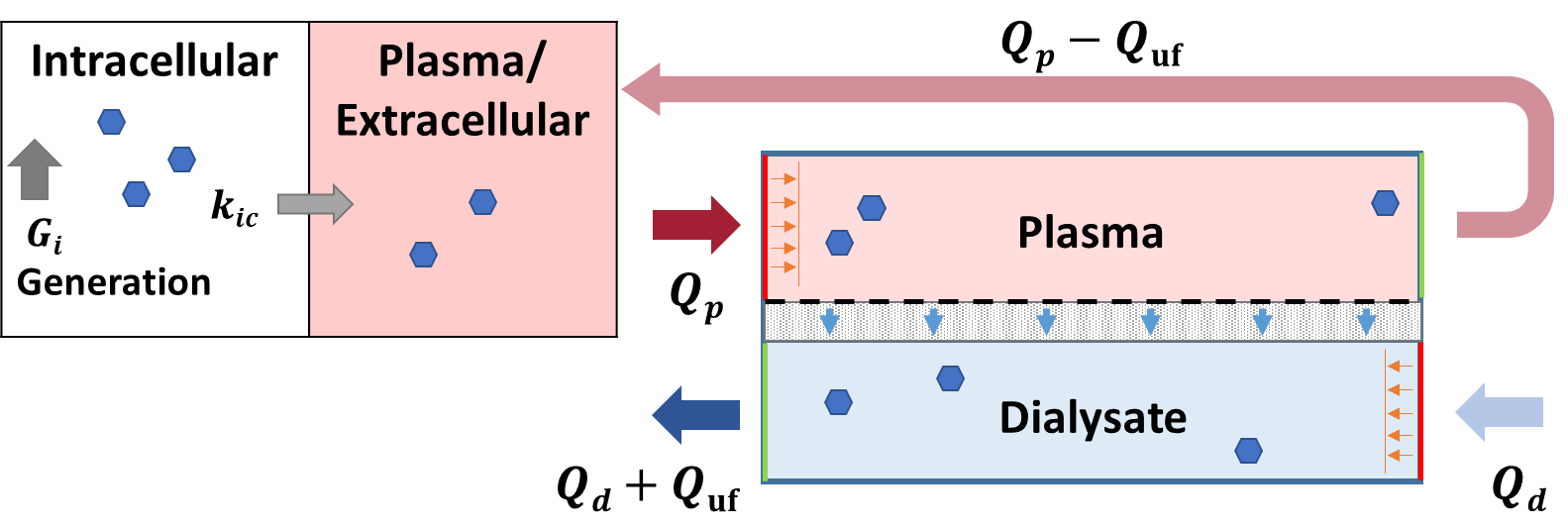


**Figure S10.** Cartoon depicting the compartment model used for urea.

To determine the overall permeance, we assume that urea permeance is 2 times higher than IS permeance, based on their relative diffusivity: urea diffusivity of 1.2×10^-9^ m^2^s and IS diffusivity of ~5×10^-10^ m^2^s in the plasma, determined from our experiments on IS (unpublished), which we cross-checked against L-tryptophan and other indole-derivatives with similar size and structure.^4,12,20,22,29^ Clinical studies indicate that the KoA observed for urea was ~750 mL min^-1^ for a 1.96 m^2^ dialyzer. ^1^ This corresponds to $P_{df,urea}$ ~ 6.4×10^-6^ m s^-1^, which is 2 times of $P_{df,IS}$ ~
3×10^-6^ m s^-1^, confirming that our use of 2 as the permeance difference factor is reasonable, assuming that the dominant resistance is the same for both systems.

The base case simulation at $P_{df,urea}$ = 2$P_{df,IS}$ = 6×10^-6^ m s^-1^, $A_{m}$ = 1.87 m^2^, $Q_{b}$ = 300 mL min^-1^, and $\tau$ = 4 h leads to a urea fractional net removal $f_{\Delta q_{\mathrm{net}}}$ = 64%, which corresponds to reduction ratios MRR = 0.717 (mass), RR = 0.654 (concentration), and $K_{Cl,\dot{M}}$ = 186 mL min^-1^ (clearance calculated from membrane integration). These numbers are cross-checked against several literature studies with similar operation parameters, which yield similar results with urea reduction ratio (URR) ~0.6-0.7, clearance ~160-200 mL min^-1^, suggesting that our urea compartment model should have reasonable estimation power.^1,3,7,32^

Figure S11 shows the simulation results for the urea compartment model at the base case. Because of urea’s small size and fast transport, MRR, RR, and fractional net removals are similar (i-iii). The net toxin removal tends to be slower than the removal from the plasma compartment, and MRR is higher than the concentration-based RR because of the concentrating effect from fluid removal. The removal from the intracellular compartment faces a slight delay *vs*. that from the plasma (vi *vs.* v) as it takes time for urea to transport from the former to the latter before being removed by the dialyzer. Similar to the IS case, the three clearance metrics give slightly different profiles (vii-ix), with $K_{Cl,K_{o}A}$ matching closely with $K_{Cl,\dot{M}}$ since the free fraction of urea is always 1 without protein-binding. At high permeance, all urea that enters the device is removed (DRR~1, x), and removal from the body is limited by how fast urea goes between the compartments and the blood flow rate into the dialyzer.


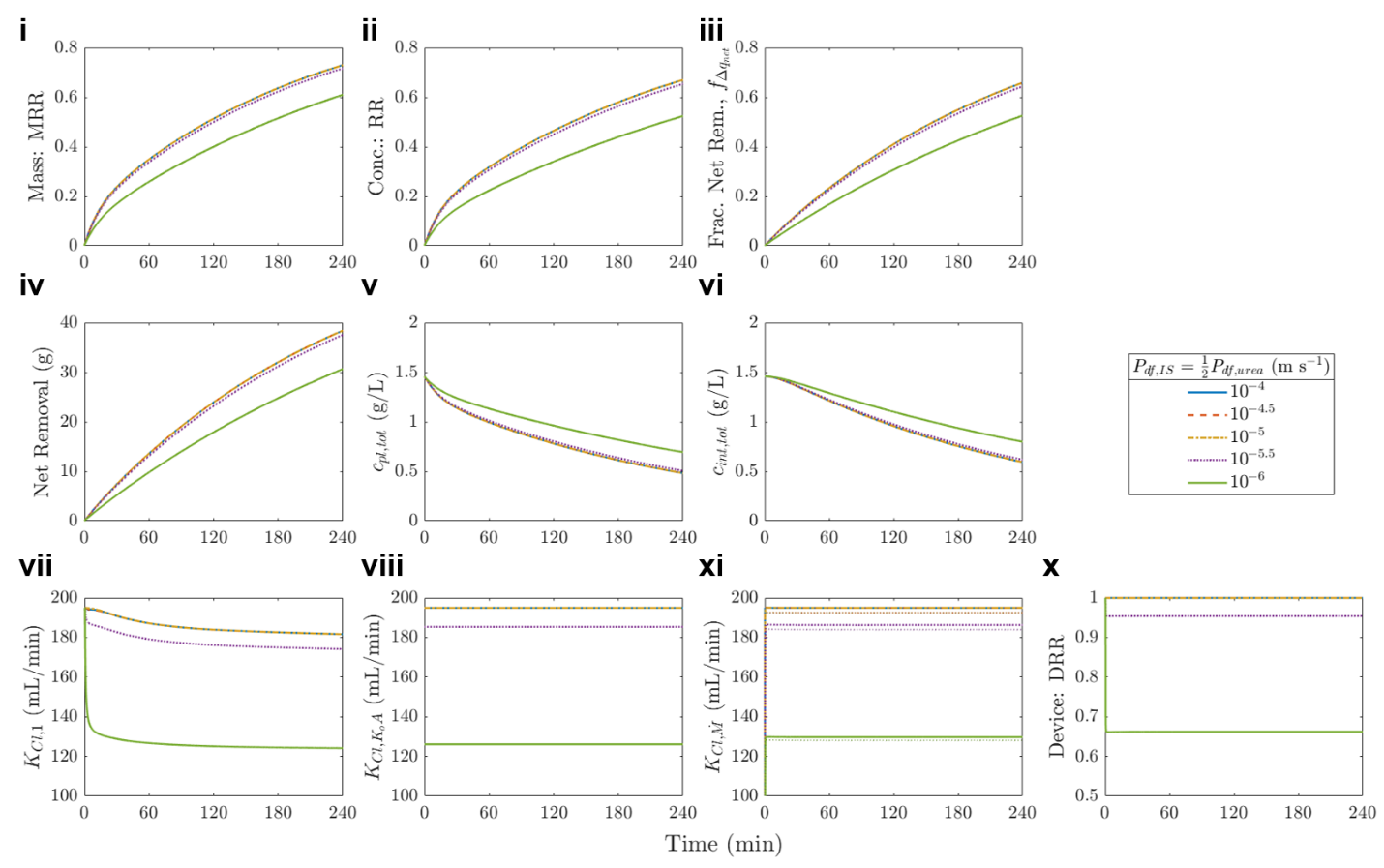


**Figure S11.** Changes in urea removal metrics over a 4 h dialysis period for various permeances. Top row: (i) Mass reduction ratios (MRR), (ii) RR, (iii) fractional net removal. Middle row: (iv) net removal, (v) plasma/extracellular concentration, (vi) intracellular concentration. Bottom row: clearances (vii) single-compartment $K_{Cl,1}$, (viii) free fraction (plasma compartment) $K_{Cl,K_{o}A}$, (xi) membrane mass transport $K_{Cl,\dot{M}}$; (xii) device removal ratio DRR.

# S9. Maximum allowable albumin loss: Implications on protein permeance and selectivity

*a. Note on membrane terminology*

Two membrane-related terms—permeance and selectivity—are commonly used to describe membrane performance. *Permeance*, $P_{\mathrm{df},i}$, reflects “how fast” a species can pass through the membrane and translates to the “throughput” of the device, and is defined via the relation:

$J_{i}=P_{\mathrm{df},i}\Delta c_{i}$ (58)

where $J_{i}$ is the diffusive molecular flux (molar flow rate per membrane area) of species $i$
[mol m^-2^s^-1^], and $\Delta c_{i}$ is the concentration gradient across the two membrane boundary layers. The permeance depends on the structure and chemistry of the membrane, and typically increases with lower thickness, lower tortuosity, and higher porosity of the membrane. Two other terms related to permeance are also used often in the literature. Membrane permeability is the permeance divided by membrane thickness, and the dialyzer mass transfer area coefficient ($K_{o}A$) is the overall mass transfer coefficient ($K_{o}=P_{\mathrm{df}}$, in which membrane permeance is one of the major components along with bulk and boundary layer mass transfer) multiplied by the overall membrane area ($A=A_{m}$). $K_{o}A$ is frequently used as an averaged description of the membrane performance in an actual dialyzer. However, decoupling the permeance from the membrane area is important to inform membrane design and engineering, and thus permeance is more commonly used in the membrane community.

*Selectivity* for species X over Y is the ratio of permeances of the two species:

$S_{X/Y}=\frac{P_{\mathrm{df},X}}{P_{\mathrm{df},Y}}$ (59)

where X is typically the species to be removed and Y is the species to be retained. Selectivity determines “how effective” the membrane is at separations. The ideal hemodialysis membrane would have high permeance and high selectivity for toxins over proteins (i.e. low protein permeance). However, there is typically a trade-off between permeance and selectivity, since larger pores would lead to higher permeance, but they could also potentially lead to higher leakage of compounds needed to be retained. One remedy is to have narrower pore size distributions, which can achieve a sharper cut-off between molecules to remove *vs.* those to retain, and thus allows for larger pore sizes that could increase molecular fluxes. Rather than selectivity, many dialyzer studies report the sieving coefficient instead, which is defined by the ISO as the “ratio of a solute concentration in the filtrate to the simultaneous concentration of the same solute in the plasma”, and is calculated through *in vitro* experiments where samples are drawn at given times at the inlet and outlet of a recirculating fluid device operated in hemodialysis/ hemofiltration modes and their concentrations are measured. Yet, depending on the flow rates, ultrafiltration rates, sampling time, test plasma, the determined sieving coefficients can show marked differences.^11^ A related quantity is the molecular weight cut-off (MWCO), which is an empirical membrane characteristic that is related to the membrane pore size and is defined as the molecular weight of molecules for which the sieving coefficient is 0.1.^39^ For membrane design, it is preferred to use membrane quantities that are independent of the flow dynamics and can be directly related to membrane structures so as to facilitate membrane engineering, so this study focuses on permeance and selectivity.

*b. Albumin loss*

In addition to the technical difficulty in achieving extremely high permeance (>3×10^-5^ m s^-1^) for practical membranes (where boundary layer resistances would start to dominate), all membranes face a tradeoff between permeance and selectivity (toxin/albumin), though the magnitude of the tradeoff depends on the separation mechanism. In engineering membranes for increased toxin permeance, the protein permeance should be kept low to avoid negative health effects. The typical recommended tolerance level is no more than 4 g albumin loss per treatment, though there is no hard evidence of physiological damage if the loss is <20 g.^30,39^ To design the appropriate dialysis membrane would require knowing the corresponding minimum selectivity required for different PBUT permeances.

The multi-compartment model allows us to determine this by estimating the maximum tolerable protein permeance for a given treatment duration $\tau$, membrane area $A_{m}$, and protein loss limit (e.g. 20 g), assuming that ultrafiltration loss is negligible compared to diffusion (since $\mathrm{Pe}_{m}=S_{\infty}Q_{\mathrm{uf}}/P_{\mathrm{df}}A_{m}\ll1$). For minimal protein loss, the concentration gradient across the membrane is almost constant for all positions and time ($\Delta c_{P}$ ≈ 40 g/L), thus by mass balance: Maximum allowable protein loss rate ≈ Protein loss limit/$\tau$ ≈ $P_{df, P,max} \Delta c_{P}A_{m}$. Rearranging, we have:

$P_{df, P,max}=\frac{Protein loss limit}{\tau\Delta c_{P} A_{m}}$ (60)

where $P_{df, P,max}$ is the maximum allowable protein permeance. The minimum toxin/protein selectivity is then $S_{toxin/P, min}=P_{df,toxin}/P_{df, P,max}$.

Figure S12 plots $P_{df, P,max}$ and $S_{toxin/P, min}$ as a function of IS net removal for different permeances, assuming a typical treatment time of 4 h. For any given PBUT removal target, higher IS permeance allows for higher albumin leakage (higher $P_{df, P,max}$) because the corresponding membrane area can be lowered. Thus, while at any given point the protein flux is higher, the overall protein loss becomes lower. The $P_{df, P,max}$ curves tend to converge at high toxin removal and high permeances, as toxin removal performance saturates due to diminishing returns (Figure S12a). The minimum required selectivity, in contrast, contains only 1 curve that describe all permeances due to the inverse relationship between $P_{df,toxin}$ and $A_{m}$ in $K_{o}A$ (Figure S12b). For an IS removal of 0.15 g (22%), 20 g protein loss, and IS permeance of 10^-5^ m s^-1^, the corresponding $P_{df, P,max}$ ≈ 5.9 × 10^-8^ m s^-1^ and $S_{IS/P, min}$ ≈ 170. Similar analyses could be performed for different loss limits and desired PBUT removal, but the values above provide a rough guidance for membrane design.


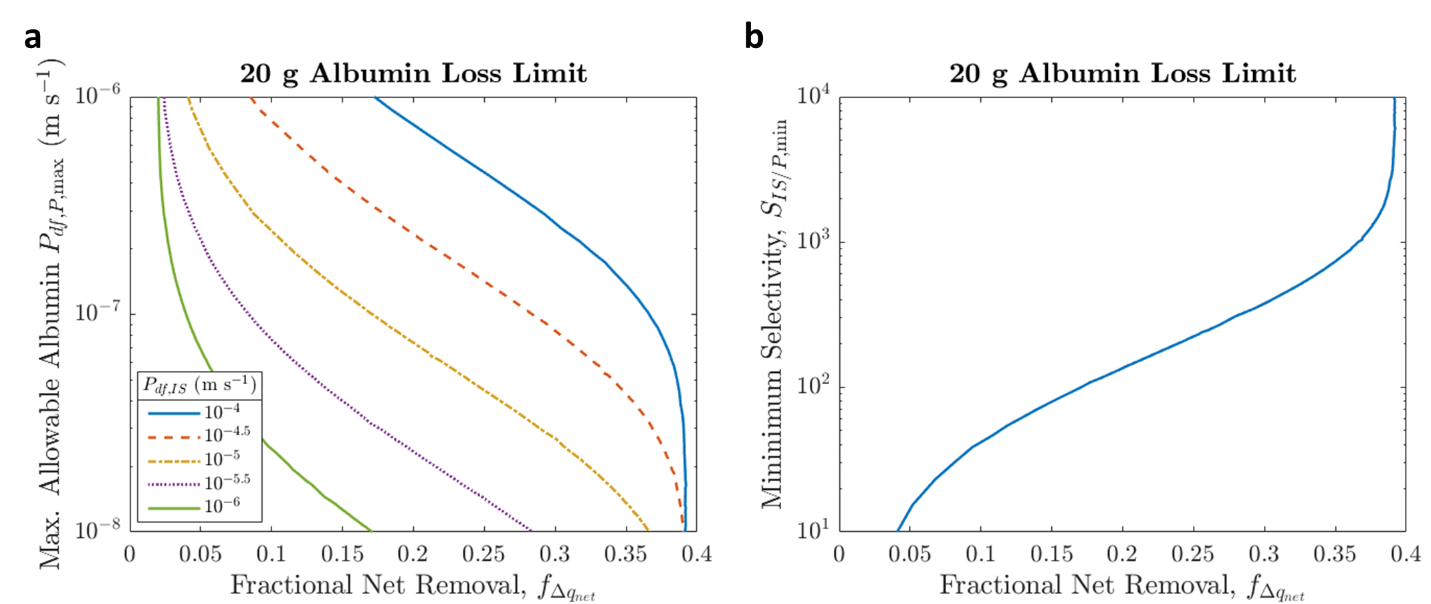


**Figure S12.** (a) Maximum allowable albumin permeance and (b) minimum IS/P selectivity for different IS net removal values and various IS permeances, assuming a maximum allowable albumin loss of 20 g. One can estimate the permeance or selectivity for other protein loss limits via multiplying or dividing the values by (desired protein loss limit)/20 g respectively.

# S10. Additional device model figures

*Device concentration profile for base case* ($Q_{b,\mathrm{in}}$: 300, $Q_{d,\mathrm{in}}$: 800, $Q_{\mathrm{uf}}$: 10 mL min^-1^; $P_{\mathrm{df}}$: 3×10^-6^ m s^-1^)

The device model solves a set of coupled differential equations for the counter-current concentration profile for the blood and dialysate channels. As toxins are removed from the blood, the free toxin concentration drops while the free protein concentration rises. However, because of the strong affinity, the bounded-toxin concentration remains high. While the actual amount of bounded-toxin in the blood falls, there is a concentrating effect as ultrafiltration removes fluid.

**
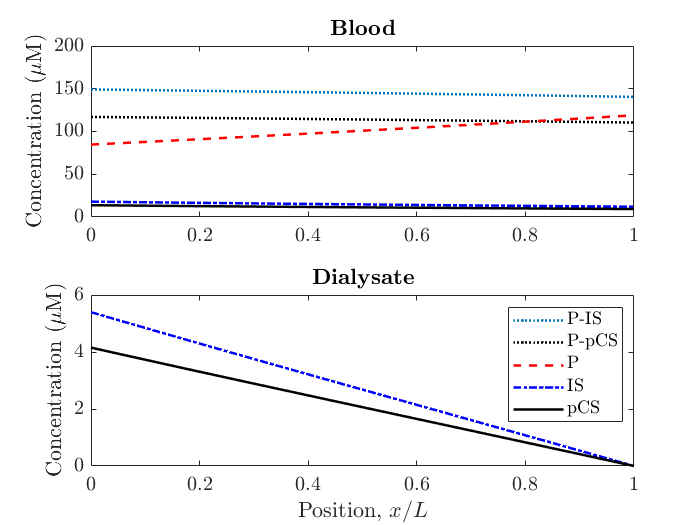
**

**Figure S13.** Device concentration profile assuming no protein leakage. The blood inlet is on the left and the dialysate inlet is on the right. The dialysate concentration is set to be 0 at the inlet.

*Dialysate outlet concentration for PBUT vs. non-bound toxins*

Here, we examine how the dialysate outlet toxin concentration $c_{d,\mathrm{out},i}$ changes as device/process parameters change. For increasing $\tilde{Q}_{d/p}$, DRR increases (Figure 2, main text) while outlet concentration goes down because an increase in $\tilde{Q}_{d/p}$ (i.e. dialysate flow rate) is larger than the decrease in concentration, leading to an overall increase in toxin removal out of the device. At low $\tilde{Q}_{d/b}$, $c_{d,\mathrm{out},i}$ is saturated at the inlet free concentration of 17 μM (set as scaled concentration of 1) since for diffusion-driven flux the dialysate concentration cannot exceed that of the blood. The PBUT scaled concentrations also tend to be slightly higher than the non-protein bounded toxins for the same parameters, as there is replenishment from the protein-bounded toxins for the former and scaling is done using the inlet free concentration.


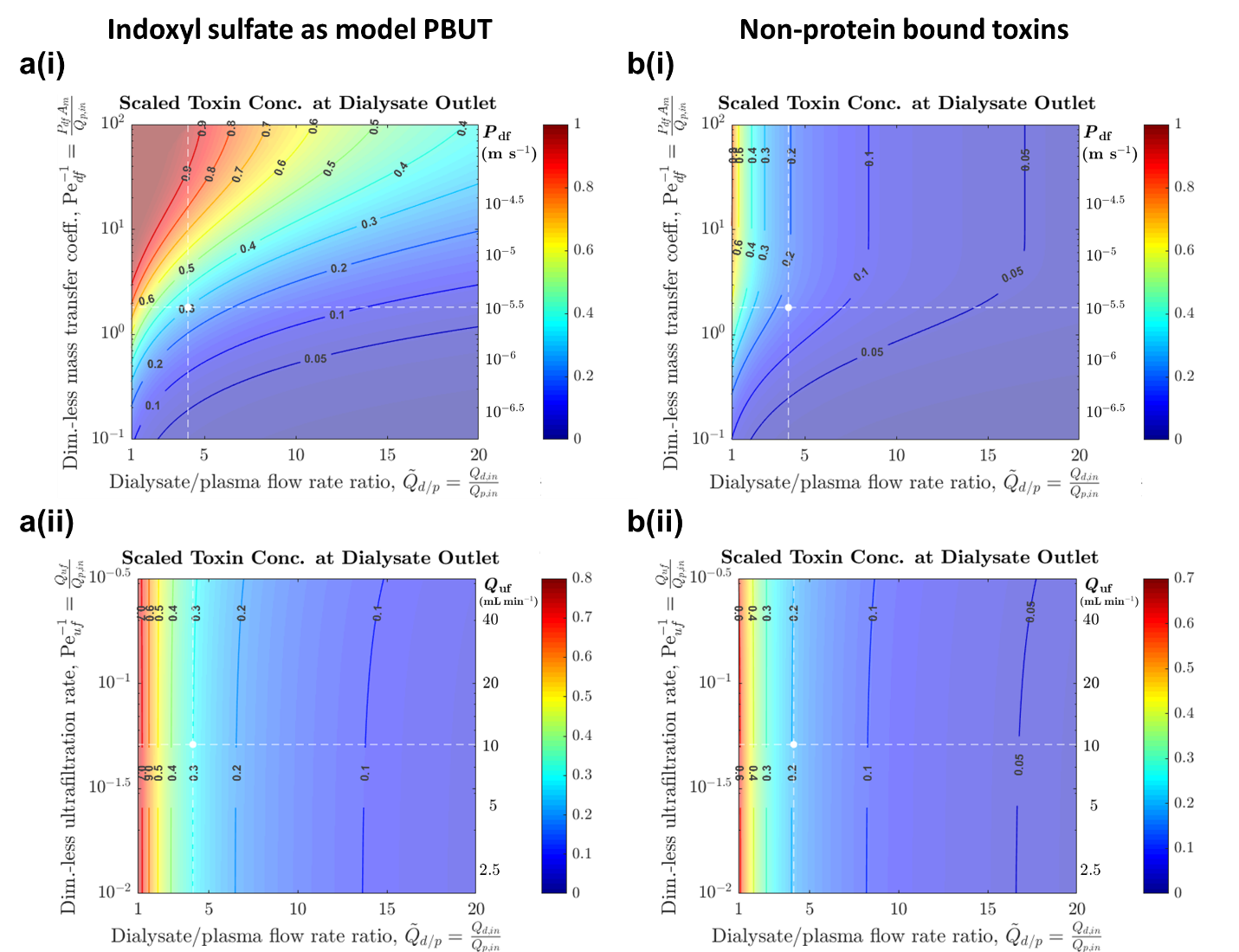


**Figure S14.** Effect of increasing dialysate flow rate $Q_{d,\mathrm{in}}$, overall permeance $P_{\mathrm{df}}$, or ultrafiltration rate $Q_{\mathrm{uf}}$ on toxin removal for (a) PBUTs and (b) Non-PBUTs, e.g. urea, creatinine. The contour levels and color denote the scaled toxin concentration, where a scale of 1 is set as the inlet free toxin concentration. Dimensionless parameters are plotted. All plots: $x$ = dialysate/blood flow ratio. Top panel (i): $y$ = $\mathrm{Pe}_{df,IS}^{-1}\propto P_{\mathrm{df},\mathrm{IS}}$, constant $Q_{\mathrm{uf}}$ = 10 mL min^-1^. Bottom panel (ii): $y$ = $\mathrm{Pe}_{\mathrm{uf}}^{-1}\propto$ $Q_{\mathrm{uf}}$, constant $P_{\mathrm{df},\mathrm{IS}}$ = 3×10^-6^ m s^-1^. Baseline parameter levels for IS are denoted by the white/black dot and dashed lines for (a), and the same dot and lines are presented in (b).

# S11. References

1. Bhimani, J. P., R. Ouseph, and R. A. Ward. Effect of Increasing Dialysate Flow Rate on Diffusive Mass Transfer of Urea, Phosphate and β2-Microglobulin During Clinical Haemodialysis. *Nephrology Dialysis Transplantation* 25:3990–3995, 2010.

2. Busher, J. T. Serum Albumin and Globulin. *Clinical Methods: The History, Physical, and Laboratory Examination* 3:497–499, 1990.

3. Clark, W. R., J. K. Leypoldt, L. W. Henderson, B. A. Mueller, M. K. Scott, and E. F. Vonesh. Quantifying the Effect of Changes in the Hemodialysis Prescription on Effective Solute Removal with a Mathematical Model. *J. Am. Soc. Nephrol.* 10:601–609, 1999.

4. Colton, C. K., K. A. Smith, E. R. Merrill, and S. Friedman. Diffusion of Urea in Flowing Blood. *AIChE Journal* 17:800, 1971.

5. Cornelis, T., S. Eloot, R. Vanholder, G. Glorieux, F. M. van der Sande, J. L. Scheijen, K. M. Leunissen, J. P. Kooman, and C. G. Schalkwijk. Protein-Bound Uraemic Toxins, Dicarbonyl Stress and Advanced Glycation End Products in Conventional and Extended Haemodialysis and Haemodiafiltration. *Nephrology Dialysis Transplantation* 30:1395–1402, 2015.

6. Dupire, J., M. Socol, and A. Viallat. Full dynamics of a red blood cell in shear flow. *Proc Natl Acad Sci U S A* 109:20808–20813, 2012.

7. Eloot, S., W. van Biesen, A. Dhondt, H. van de Wynkele, G. Glorieux, P. Verdonck, and R. Vanholder. Impact of Hemodialysis Duration on the Removal of Uremic Retention Solutes. *Kidney Int* 73:765–770, 2008.

8. Eloot, S., D. Schneditz, and R. Vanholder. What Can the Dialysis Physician Learn from Kinetic Modelling Beyond Kt/Vurea? *Nephrology Dialysis Transplantation* 27:4021–4029, 2012.

9. European Work Group on Uremic Toxins (EUTox). Uremic Solutes Database, 2018, at <http://eutoxdb.odeesoft.com/soluteList.php>

10. Gotch, F. A., and J. A. Sargent. A Mechanistic Analysis of the National Cooperative Dialysis Study (NCDS). *Kidney Int* 28:526–534, 1985.

11. Hulko, M., U. Haug, J. Gauss, A. Boschetti-de-Fierro, W. Beck, and B. Krause. Requirements and Pitfalls of Dialyzer Sieving Coefficients Comparisons. *Artif Organs* 42:1164–1173, 2018.

12. Kidambi, P. R., D. Jang, J. C. Idrobo, M. S. H. Boutilier, L. Wang, J. Kong, and R. Karnik. Nanoporous Atomically Thin Graphene Membranes for Desalting and Dialysis Applications. *Advanced Materials* 29: 1700277, 2017.

13. Krieter, D. H., A. Hackl, A. Rodriguez, L. Chenine, H. L. Moragues, H. D. Lemke, C. Wanner, and B. Canaud. Protein-Bound Uraemic Toxin Removal in Haemodialysis and Post-Dilution Haemodiafiltration. *Nephrology Dialysis Transplantation* 25:212–218, 2010.

14. Luo, F. J. G., K. P. Patel, I. O. Marquez, N. S. Plummer, T. H. Hostetter, and T. W. Meyer. Effect of Increasing Dialyzer Mass Transfer Area Coefficient and Dialysate Flow on Clearance of Protein-Bound Solutes: A Pilot Crossover Trial. *American Journal of Kidney Diseases* 53:1042–1049, 2009.

15. Madero, M., K. B. Cano, I. Campos, X. Tao, V. Maheshwari, J. Brown, B. Cornejo, G. Handelman, S. Thijssen, and P. Kotanko. Removal of Protein-Bound Uremic Toxins during Hemodialysis Using a Binding Competitor. *Clinical Journal of the American Society of Nephrology:CJASN* 14:394, 2019.

16. Maheshwari, V., S. Thijssen, X. Tao, D. H. Fuertinger, F. Kappel, and P. Kotanko. In Silico Comparison of Protein-Bound Uremic Toxin Removal by Hemodialysis, Hemodiafiltration, Membrane Adsorption, and Binding Competition. *Sci Rep* 9:909, 2019.

17. Maheshwari, V., S. Thijssen, X. Tao, D. Fuertinger, F. Kappel, and P. Kotanko. A Novel Mathematical Model of Protein-Bound Uremic Toxin Kinetics During Hemodialysis. *Sci Rep* 7:10371, 2017.

18. Martinez, A. W., N. S. Recht, T. H. Hostetter, and T. W. Meyer. Removal of P-cresol Sulfate by Hemodialysis. *Journal of the American Society of Nephrology* 16:3430–3436, 2005.

19. Meyer, T. W., E. C. Leeper, D. W. Bartlett, T. A. Depner, Y. Z. Lit, C. R. Robertson, and T. H. Hostetter. Increasing Dialysate Flow and Dialyzer Mass Transfer Area Coefficient to Increase the Clearance of Protein-Bound Solutes. *Journal of the American Society of Nephrology* 15:1927–1935, 2004.

20. Nanne, E. E., C. P. Aucoin, and E. F. Leonard. Shear Rate and Hematocrit Effects on the Apparent Diffusivity of Urea in Suspensions of Bovine Erythrocytes. *ASAIO (American Society for Artificial Internal Organs) Journal* 56:151–156, 2010.

21. National Institute of Diabetes and Digestive and Kidney (NIDDK). Hemodialysis Dose & Adequacy. NIH Publication No. 09–4556, 2009.

22. Robinson, D., J. E. Anderson, and J.-L. Lin. Measurement of Diffusion Coefficients of Some Indoles and Ascorbic Acid by Flow Injection Analysist. *J. Phys. Chem* 94:1003–1005, 1990.

23. Ronco, C., A. Brendolan, C. Crepaldi, M. Rodighiero, and M. Scabardi. Blood and Dialysate Flow Distributions in Hollow-Fiber Hemodialyzers Analyzed by Computerized Helical Scanning Technique. *J. Am. Soc. Nephrol.* 13:53–61, 2002.

24. Ronco, C., and W. R. Clark. Haemodialysis Membranes. *Nat Rev Nephrol* 14:394–410, 2018.

25. Schneditz, D., J. C. van Stone, and J. T. Daugirdas. A Regional Blood Circulation Alternative to In-series Two Compartment Urea Kinetic Modeling. *ASAIO (American Society for Artificial Internal Organs) Journal* 39:M573–M577, 1993.

26. de Smet, R., A. Dhondt, S. Eloot, F. Galli, M. A. Waterloos, and R. Vanholder. Effect of the super-flux cellulose triacetate dialyser membrane on the removal of non-protein-bound and protein-bound uraemic solutes. *Nephrology Dialysis Transplantation* 22:2006–2012, 2007.

27. Smye, S. W., and E. J. Will. A Mathematical Analysis of a Two-Compartment Model of Urea Kinetics. *Phys. Med. Biol* 40:2005–2014, 1995.

28. Sridharan, S., E. Vilar, J. Berdeprado, and K. Farrington. Energy Metabolism, Body Composition, and Urea Generation Rate in Hemodialysis Patients. *Hemodialysis International* 17:502–509, 2013.

29. Steiner, C. A. Mass Transfer of Urea through Blood. *Ann Biomed Eng* 9:217–225, 1981.

30. Storr, M., and R. A. Ward. Membrane Innovation: Closer to Native Kidneys. *Nephrology Dialysis Transplantation* 33:iii22–iii27, 2018.

31. Tan, J., A. Thomas, and Y. Liu. Influence of Red Blood Cells on Nanoparticle Targeted Delivery in Microcirculation. *Soft Matter* 8:1934–1946, 2012.

32. Vartia, A. Urea Concentration and Haemodialysis Dose. *ISRN Nephrol* 2013: 341026, 2013.

33. Velde, C. vander, and E. F. Leonard. Theoretical Assessment of the Effect of Flow Maldistributions on Mass Transfer Efficiency of Artificial Organs. *Med. & Biol. Eng. & Comput.* 23:224–229, 1985.

34. Viaene, L., P. Annaert, H. de Loor, R. Poesen, P. Evenepoel, and B. Meijers. Albumin is the main plasma binding protein for indoxyl sulfate and p-cresyl sulfate. *Biopharm Drug Dispos* 34:165–175, 2013.

35. Villarroel, F., E. Klein, and F. Holland. Solute Flux in Hemodialysis and Hemofiltration Membranes. *Trans. Amer. Soc. Artif. Int. Organs* 23:225–232, 1977.

36. Walther, J. L., D. W. Bartlett, W. Chew, C. R. Robertson, T. H. Hostetter, and T. W. Meyer. Downloadable computer models for renal replacement therapy. *Kidney Int* 69:1056–1063, 2006.

37. Watanabe, H., T. Noguchi, Y. Miyamoto, D. Kadowaki, S. Kotani, M. Nakajima, S. Miyamura, Y. Ishima, M. Otagiri, and T. Maruyama. Interaction between Two Sulfate-Conjugated Uremic Toxins, p -Cresyl Sulfate and Indoxyl Sulfate, during Binding with Human Serum Albumin. *Drug Metab. Dispos.* 40:1423–1428, 2012.

38. Yu, S., M. Schuchardt, M. Tolle, M. van der Giet, W. Zidek, J. Dzubiella, and M. Ballau. Interaction of human serum albumin with uremic toxins: a thermodynamic study. *RSC Adv.* 7:27913–27922, 2017.

39. Zweigart, C., A. Boschetti-de-Fierro, M. Hulko, L. G. Nilsson, W. Beck, M. Storr, and B. Krause. Medium Cut-off Membranes - Closer to the Natural Kidney Removal Function. *International Journal of Artificial Organs* 40:328–334, 2017.

40. Zydney, A. L. Bulk Mass Transport Limitations during High‐Flux Hemodialysis. *Artif Organs* 17:919–924, 1993.
